# The retrograde signaling regulator ANAC017 recruits the MKK9–MPK3/6, ethylene, and auxin signaling pathways to balance mitochondrial dysfunction with growth

**DOI:** 10.1093/plcell/koac177

**Published:** 2022-06-16

**Authors:** Cunman He, Lim Chee Liew, Lingling Yin, Mathew G Lewsey, James Whelan, Oliver Berkowitz

**Affiliations:** Department of Animal, Plant and Soil Science, La Trobe University, Bundoora, Victoria 3086, Australia; ARC Centre of Excellence in Plant Energy Biology, La Trobe University, Bundoora, Victoria 3086, Australia; Department of Animal, Plant and Soil Science, La Trobe University, Bundoora, Victoria 3086, Australia; Department of Animal, Plant and Soil Science, La Trobe University, Bundoora, Victoria 3086, Australia; Department of Animal, Plant and Soil Science, La Trobe University, Bundoora, Victoria 3086, Australia; Department of Animal, Plant and Soil Science, La Trobe University, Bundoora, Victoria 3086, Australia; ARC Centre of Excellence in Plant Energy Biology, La Trobe University, Bundoora, Victoria 3086, Australia; Department of Animal, Plant and Soil Science, La Trobe University, Bundoora, Victoria 3086, Australia; ARC Centre of Excellence in Plant Energy Biology, La Trobe University, Bundoora, Victoria 3086, Australia

## Abstract

In plant cells, mitochondria are ideally positioned to sense and balance changes in energy metabolism in response to changing environmental conditions. Retrograde signaling from mitochondria to the nucleus is crucial for adjusting the required transcriptional responses. We show that ANAC017, the master regulator of mitochondrial stress, directly recruits a signaling cascade involving the plant hormones ethylene and auxin as well as the MAP KINASE KINASE (MKK) 9–MAP KINASE (MPK) 3/6 pathway in *Arabidopsis thaliana*. Chromatin immunoprecipitation followed by sequencing and overexpression demonstrated that ANAC017 directly regulates several genes of the ethylene and auxin pathways, including *MKK9*, *1-AMINO-CYCLOPROPANE-1-CARBOXYLATE SYNTHASE 2*, and *YUCCA 5*, in addition to genes encoding transcription factors regulating plant growth and stress responses such as BASIC REGION/LEUCINE ZIPPER MOTIF (bZIP) 60, bZIP53, ANAC081/ATAF2, and RADICAL-INDUCED CELL DEATH1. A time-resolved RNA-seq experiment established that ethylene signaling precedes the stimulation of auxin signaling in the mitochondrial stress response, with a large part of the transcriptional regulation dependent on ETHYLENE-INSENSITIVE 3. These results were confirmed by mutant analyses. Our findings identify the molecular components controlled by ANAC017, which integrates the primary stress responses to mitochondrial dysfunction with whole plant growth via the activation of regulatory and partly antagonistic feedback loops.

## Teaser

The molecular components controlled by the transcription factor ANAC017, which integrates primary stress responses to mitochondrial dysfunction with whole plant growth, are identified in Arabidopsis.

IN A NUTSHELL **Background:** Mitochondria help maintain the energy status of plant cells, act as sensors for cellular homeostasis, and are the origins of a signaling pathway termed mitochondrial retrograde signaling. These functions support the acclimation of plants to environmental changes and adverse growth conditions. Retrograde signaling directly controls gene expression in response to external and internal stimuli and connects mitochondrial function with other organelles, especially chloroplasts. While the integration of these pathways is crucial for plant growth and development, the associated molecular components and their functions are largely unknown. The transcription factor (TF) ANAC017 is a master regulator of mitochondrial dysfunction, and several plant hormones, especially auxin and ethylene, contribute to the overall process.

**Question:** What are the primary targets of ANAC017? Which downstream signaling cascade leads to changes in gene expression, phytohormone levels, and ultimately plant growth?

**Findings:** We provide mechanistic insight into how ANAC017 recruits ethylene, auxin, and the MKK9–MPK3/6 pathways in *Arabidopsis thaliana*. Time-resolved RNA-seq revealed an *ETHYLENE INSENSITIVE3-*dependent, early activation of key genes involved in ethylene signaling and biosynthesis, which promotes the mitochondrial signaling pathway. The subsequent activation of auxin biosynthesis, transport, and conjugation inhibits the retrograde pathway together with RADICAL-INDUCED CELL DEATH1. Several other TFs targeted by ANAC017 were determined, including bZIP60, bZIP53, and ANAC081/ATAF2, which control growth-related processes such as senescence and the unfolded protein response. Therefore, ANAC017 is the upstream activator of regulatory feedback loops that coordinate mitochondrial function with whole plant growth.

**Next steps:** Manipulating plant signaling pathways to improve crop performance has been challenging. Transgenic approaches often lead to reduced growth or yield penalties due to the activation of stress response pathways under normal conditions. A better understanding of the interconnected regulatory pathways and TFs such as ANAC017 will help separate beneficial from detrimental effects in translational research approaches.

## Introduction

Mitochondria play a central role in plant energy homeostasis and adjust metabolism to the prevailing growth conditions through oxidative phosphorylation and the central role of the tricarboxylic acid cycle. These functions are also crucial for plants to respond to changes in environmental settings and acclimate to adverse growth conditions. In accordance with this observation, mitochondria also sense stress, allowing the integration with the overall cellular response largely driven from the nucleus. Mitochondria relay their status to the nucleus via a pathway termed the mitochondrial retrograde response (MRR) that adjusts the expression of nuclear genes to optimize mitochondrial function. This is also coordinated with a function paralleled by chloroplasts and partly overlapping signaling pathways that are together also parts of stress signaling pathways (Crawford et al., 2018; Wang et al., 2018; Dopp et al., 2021). While nonoptimal conditions passively slow plant growth as a consequence of reduced energy supply and/or redirection of metabolic resources, the activation of such signaling pathways also triggers a reduction in growth. This is often an unwanted side-effect of biotechnological approaches to improve plant stress tolerance by genetically modifying signaling pathways because the underlying and interconnected regulatory networks are poorly understood (Zhang et al., 2020).

Triggering mitochondrial dysfunction, for example by using chemical inhibitors of mitochondrial electron transport such as antimycin A (AA; complex III) and myxothiazol (MT, complex III), or by mutating genes, has allowed for the identification of many stress-responsive genes through genetic and transcriptomic approaches (Wang et al., 2018). This led to the early identification of 24 mitochondrial dysfunction stimulon (*MDS*) genes based on their high induction when mitochondrial function was impaired (De Clercq et al., 2013). This work, together with a forward genetic screen for regulators of the mitochondrial stress marker gene *ALTERNATIVE OXIDASE 1A* (*AOX1A*), also part of the MDS, revealed several key transcription factors (TFs) involved in this regulation in *A.thaliana* (Ng et al., 2013). These TFs, ANAC013 and ANAC017, belong to the Arabidopsis NAC (ANAC) domain containing family located at the endoplasmic reticulum (ER) membrane, and their targeted *MDS* genes share a common binding motif (De Clercq et al., 2013; Ng et al., 2013). This response directly regulates the expression of hundreds of genes, as evidenced by attenuated transcriptional responses in the corresponding mutant lines and increased expression of genes in *ANAC017* overexpressing lines (Ng et al., 2013; Van Aken et al., 2016; Meng et al., 2019). However, it is unknown how many of these genes are directly targeted by ANAC013 or ANAC017, and which genes are further downstream in the transcriptional cascade. A central role of ANAC017 is also evident from its function in regulating some chloroplast retrograde responses, and it is therefore a convergence point for organellar signaling pathways (Van Aken et al., 2016; Meng et al., 2019). Correspondingly, the mutation of *ANAC017* leads to increased sensitivity to combined light/drought stress, submergence, and reductive stress (Ng et al., 2013; Meng et al., 2019; Fuchs et al., 2022). ANAC017 also regulates plant growth, as overexpression and mutation leads to reduced and increased growth under nonlimiting conditions, respectively, indicating a role of ANAC017 in balancing mitochondrial function in response to stress with plant growth (Ng et al., 2013; Meng et al., 2019, 2020). However, the mechanisms underlying how ANAC017 exerts these diverse roles have not been resolved.

A role has been established for only a few *MDS* genes and their encoded proteins in mitigating mitochondrial stress, while for many, their immediate role in this process is not directly obvious from their molecular function. The MDS protein AOX1A is part of the alternative pathway in the electron transport chain that prevents its detrimental over-reduction under adverse conditions, while the alternative subcellular localization of At12Cys suggests it might function as a signaling component (Clifton et al., 2006; Wang et al., 2016). Two other *MDS* genes, *ATP-BINDING CASSETTE B4* (*ABCB4*) and *URIDINE DIPHOSPHATE GLYCOSYLTRANSFERASE 74E2* (*UGT74E2*), are directly linked to auxin homeostasis by encoding an auxin transporter and auxin conjugase, respectively (Noh et al., 2001; Tognetti et al., 2010). A role for auxin in regulating mitochondrial function was also established by a screen for Arabidopsis mutants with impaired MRR, which identified the auxin transporters BIG, PINFORMED (PIN) 1, and ABCB19 as well as ASYMETRIC LEAVES 1, the latter being involved in the establishment of auxin gradients in the leaf. Together, these findings provided evidence for a feedback loop and antagonistic relationship between the MRR and auxin (Ivanova et al., 2014; Kerchev et al., 2014).

Two *MDS* genes have links to the ethylene pathway. The class VII ethylene response factor (ERF) HYPOXIA-RESPONSIVE ERF 2 (HRE2)/ERF71 participates in the oxygen status-sensing N-end rule pathway and modulates ethylene responses under hypoxia (Licausi et al., 2010; Gasch et al., 2016). The gene *AT5G43450* encodes an uncharacterized member of the 2-oxoglutarate (2OG) and Fe(II)-dependent oxygenase family and is annotated as an ACO-like gene in TAIR10 due to its sequence similarity to the ethylene biosynthesis enzyme 1-AMINO-CYCLOPROPANE-1-CARBOXYLIC ACID (ACC) oxidase (ACO; Trentmann and Kende, 1995; Vandenbussche et al., 2003). Ethylene modulates the proteotoxic response when impaired mitochondrial function results in the accumulation of misfolded proteins (Wang and Auwerx, 2017; Kacprzak et al., 2020). Inhibited mitochondrial translation in a mutant deficient in the organellar polymerase RPOTmp leads to an AOX1A-dependent phenotype reminiscent of the triple-response observed for ethylene-treated wild-type plants (Merendino et al., 2020). Ethylene also activates the MRR to increase reactive oxygen species production during seed germination to break dormancy (Jurdak et al., 2021). In addition, the transcriptional responses to hypoxia and submergence are largely controlled by ethylene signaling, but also by MRR regulators such as ANAC017, and substantially overlap with AA-induced mitochondrial dysfunction, suggesting a role for ethylene in the MRR pathway (Wagner et al., 2018; Meng et al., 2020). A mechanistic understanding for an ethylene-modulated MRR pathway is currently scarce. Kacprzak et al. (2020) showed a stimulation of AA-induced gene expression in an ethylene-overproducing mutant (*eto1-1*), while mutants for the signaling components ETHYLENE INSENSITIVE 2 and MITOGEN-ACTIVATED PROTEIN KINASE (MPK) 6 showed no difference from the wild-type. The authors concluded that ethylene promotes the MRR independently of ANAC017. Results from RNA-sequencing (RNA-seq) data of laser capture microdissected leaf tissue, however, suggested that a more localized interaction of ethylene and auxin influences AA-induced transcription (Berkowitz et al., 2021).

Although together these results establish ANAC017 as a master regulator of mitochondrial signaling and provide evidence that auxin and ethylene play a role in regulating the MRR, there is no clear understanding of how these pathways are activated or coordinated. Here, by time-resolved RNA-seq, chromatin immunoprecipitation followed by sequencing (ChIP-seq), and mutant analyses, we provide direct evidence that ANAC017 drives the primary response by upregulating stress-mitigating genes such as *AOX1A* in Arabidopsis. In parallel, ANAC017 also directly activates regulatory cascades involving the MAP KINASE KINASE (MKK9)–MPK3/6 pathway, its own repressor RADICAL-INDUCED CELL DEATH1 (RCD1), as well as ethylene and auxin signaling to provide antagonistic feedback loops to balance these responses. In addition, by targeting downstream TFs, such as ANAC081/ATAF2, BASIC REGION/LEUCINE ZIPPER MOTIF (bZIP60), or bZIP53, ANAC017 co-ordinates mitochondrial function to balance stress responses with plant growth.

## Results

### An *AOX1A* co-expression network identifies genes related to ethylene signaling

**Figure 1:**
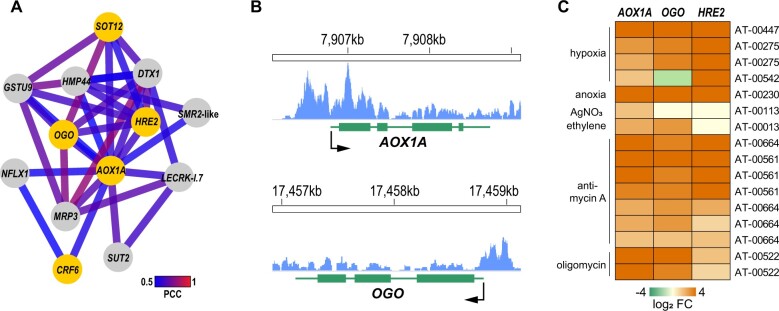
A co-expression network of *AOX1A* involves ethylene signaling-related genes. A, Co-expression network analysis of *AOX1A* with the CoNekT tool kit (Proost and Mutwil, 2018) identified 12 genes with highly correlated gene expression patterns. In the resulting network, two genes close to *AOX1A* were related to ethylene signaling, that is, the ERF *ERF71/HRE2* and *OGO/AT5G43450*. Edge color represents the Pearson correlation coefficient (PCC). Genes of the MDS are represented by orange circles (De Clercq et al., 2013). B, HRE2 binds to the promoters of *AOX1A* and *OGO*. Shown are genome browser views of the ChIP-seq read coverage at the promoters of the two genes. ChIP-seq data were downloaded from the NCBI SRA database (SRR8234099) and aligned to the TAIR10 Arabidopsis genome release. Both genes were among the significant peak calls, as identified previously by (Lee and Bailey-Serres, 2019). C, Heatmap showing induced expression of *AOX1A*, *OGO*, and *HRE2* in various experiments involving ethylene-dependent signaling (hypoxia, anoxia), altering ethylene tissue-concentrations (ethylene gas, AgNO_3_) or induce mitochondrial stress (AA, oligomycin). Publicly available gene expression data were retrieved using the Genevestigator platform, and data set IDs are indicated (Hruz et al., 2008).

The treatment of plants with AA to inhibit complex III and induce mitochondrial dysfunction has greatly helped to elucidate mechanisms in mitochondrial retrograde signaling (Wang et al., 2020). This allowed for the identification of key genes involved in this response, such as the mitochondrial stress marker gene *AOX1A* (Clifton et al., 2005). Using the CoNekT tool, which incorporates 913 RNA-seq data sets of Arabidopsis for a variety of tissues and developmental stages (Proost and Mutwil, 2018), we generated a co-expression neighborhood network for *AOX1A*. This identified 12 genes based on stringent parameters using a Pearson correlation coefficient above 0.5 and a highest reciprocal rank of above 100 (Figure 1A). Only 5 of the 24 *MDS* genes, which are highly upregulated by AA treatment (De Clercq et al., 2013), were part of this network. Of these, *HRE2/ERF71* and *AT5G43450* had the closest connection to *AOX1A* in this network (Figure 1A). Interestingly, both genes have an association to ethylene signaling. HRE2 is a class VII ERF involved in the oxygen-sensing N-end rule pathway and hypoxia responses (Gasch et al., 2016). The gene *AT5G43450*, encoding a 2OG and Fe(II)-dependent oxygenase that we named OGO, shares sequence similarity with the ethylene biosynthesis enzyme ACC ACO (Trentmann and Kende, 1995; Vandenbussche et al., 2003).

Additional evidence for a role of ethylene in directly connecting the three genes comes from a ChIP-seq experiment showing the binding of HRE2 to the promoters of *AOX1A* and *OGO* under hypoxia (Figure 1B; Lee and Bailey-Serres, 2019). We further retrieved the expression data of the three genes from publicly available data sets relating to ethylene (treatment with ethylene gas and the biosynthesis inhibitor AgNO_3_), hypoxia/anoxia and mitochondrial stress (AA, oligomycin) using the Genevestigator tool (Hruz et al., 2008). The three genes showed substantial upregulation across these experiments (Figure 1C), providing additional evidence for their co-expression and the involvement of ethylene in their transcriptional regulation.

**Figure 2:**
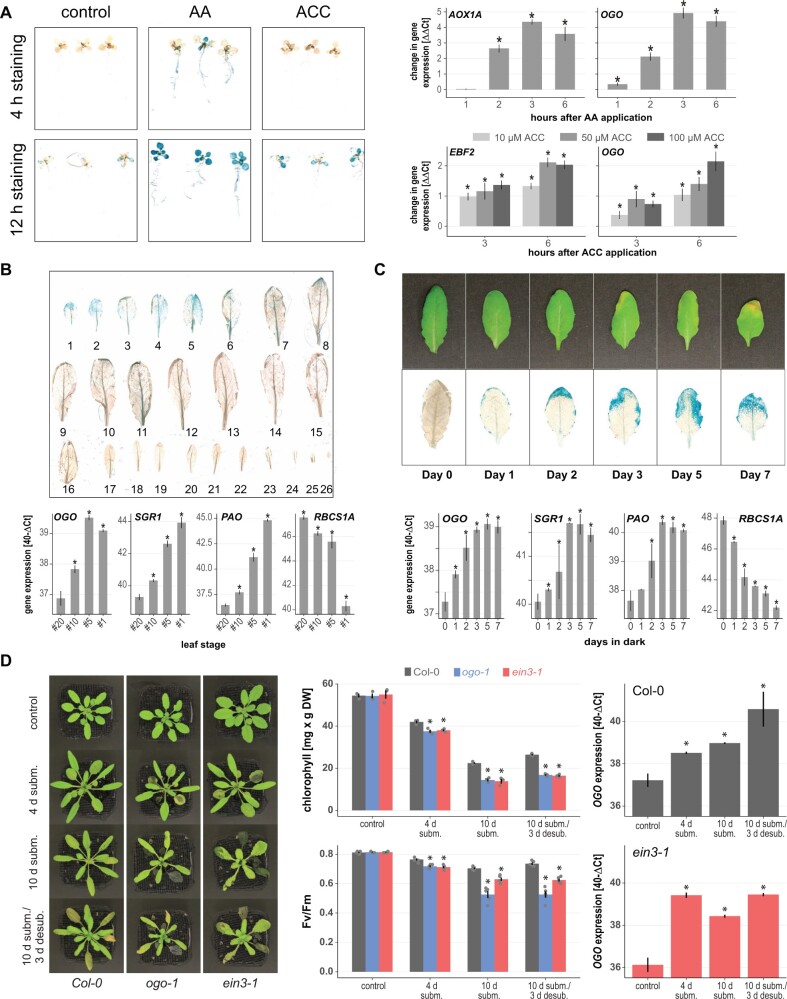
Induced expression of *OGO* by ACC, senescence, and submergence. A, *OGO* expression is upregulated by ACC and AA treatment. Left: *proOGO*-*GUS* reporter lines were treated with AA to induce mitochondrial dysfunction or ACC to increase ethylene concentrations in tissues. For both treatments, increased GUS activity was detected after 4 h and 12 h of staining, respectively. Shown are representative images of three biological replicates (separate experiments). Right: Increased expression of *OGO* after AA and ACC treatments in wild type Col-0 was also quantified by RT–qPCR. *AOX1A* and *EBF2* were used as known response genes for the treatments, respectively. Seedlings were grown on plates for 10 days, sprayed with 50-µM AA or varying ACC concentrations as indicated, and harvested 3-h post treatment. Shown are the mean ± se. Asterisks indicate statistically significant differences (*P* < 0.05, one-way (AOX1A, OGO) and two-way (EBF2, OGO) ANOVA, *n* = 3) from control (water) treatment. B, The expression of *OGO* is induced during age-dependent senescence in leaves. Top: GUS activity in rosette leaves of a representative *proOGO*-*GUS* reporter line. Leaves are numbered by the sequence of their occurrence through development. GUS staining was highest in the oldest, senescing leaves and not detectable in young leaves. Bottom: *OGO* expression was also quantified by RT–qPCR in wild-type Col-0 for the indicated leaf developmental stages. The expression of the senescence marker genes *SENESCENCE-RELATED GENE 1* (*SRG1*), *PHEOPHORBIDE A OXYGENASE* (*PAO*), and *RIBULOSE BISPHOSPHATE CARBOXYLASE SMALL CHAIN 1A* (*RBCS1A*) was also determined. Shown are the means of 40−DCt values ± se. Asterisks indicate statistically significant differences (*P* < 0.05, one-way ANOVA, *n* = 3) from youngest leaf #20. C, *OGO* promoter activity in reporter lines was also increased by dark-induced senescence. Top: Plants were kept in the dark for up to 7 days as indicated before GUS staining. Bottom: gene expression was quantified by RT–qPCR as described in (B). D, The mutant lines for *OGO* (*ogo-1*) and *EIN3* (*ein3-1*) showed a similar reduction in submergence tolerance. Shown are representative images (left), chlorophyll concentrations and maximum quantum efficiency of photosystem II (Fv/Fm) (middle panels) and the expression of *OGO* in the wild-type (Col-0), *ein3-1*, and *ogo-1* mutant lines (right) before submergence (control), after 4 and 10 days submergence, and 3 days after desubmergence as indicated. Graphs shown the mean ± se. Asterisks indicate statistically significant differences (*P < 0.05, one-way ANOVA, *n* = 3, except for Fv/Fm with *n* = 5) from the control.

To characterize *OGO* and confirm its ethylene-dependent changes in expression, we analyzed *proOGO*-*GUS* reporter lines. As expected from its MDS membership, β-glucuronidase (GUS) activity in these lines was increased by AA treatment, but also by treatment with the ethylene precursor ACC (Figure 2A, left). Consistent with this finding, AA and ACC treatments also led to increased *OGO* transcript levels, similar to the known marker genes *AOX1A* and *ETHYLENE BINDING FACTOR 2*, respectively, in wild-type Col-0, as quantified by reverse transcription–quantitative polymerase chain reaction (RT–qPCR; Figure 2A, right). Given the finding that ethylene is an important driver of leaf senescence (Kim et al., 2018), we also monitored *OGO* expression across leaf developmental stages and after dark-induced senescence (Figure 2, B and C). In the reporter lines, GUS activity was increased in the oldest leaves, while no GUS was detected in young or mature leaves (Figure 2B, upper). Similarly, *OGO* transcript levels increased with leaf age when quantified by RT–qPCR by approximately four-fold from young to old leaves (Figure 2B, lower). Upon dark-induced senescence, GUS activity in the reporter lines and *OGO* transcript levels increased with longer dark exposure (Figure 2C, upper).

Submergence is another stress for which ethylene is an important signaling component but also depends on mitochondrial signaling (Loreti et al., 2016; Meng et al., 2020). We therefore also subjected wild-type plants, an OGO knockout mutant (*ogo-1*; Supplemental Figure S1), and the *ein3-1* mutant, carrying a knockout allele of the master regulator in ethylene signaling EIN3, to submergence and desubmergence treatments. Both mutant lines showed a more severe phenotype than the wild type (Figure 2D, left). Quantification of chlorophyll concentrations and the maximum quantum yield of photosystem II (Fv/Fm) also confirmed that *ogo-1* and *ein3-1* were more strongly affected by the treatments than the wild-type (Figure 2D, middle). The expression of *OGO* also increased in Col-0 and *ein3-1*, as determined by RT–qPCR (Figure 2D, right). Taken together, these results indicate that ethylene regulates the expression of *MDS* genes and suggest a role for this hormone in regulating mitochondrial signaling.

### Transcriptional responses to mitochondrial dysfunction involve an ethylene response preceding auxin signaling

To determine if and to what extent ethylene signaling regulates mitochondrial dysfunction responses, we sampled wild-type Col-0, *ein3-1*, and *ogo-1* seedlings after spraying with the mitochondrial complex III inhibitor AA at 0, 30, 60, 120, 180, 270, and 360 min after treatment. A mock treatment using water was also performed in parallel to account for the known touch response occurring after spray treatments (Van Aken et al., 2016; Van Moerkercke et al., 2019; Xu et al., 2019). Subsequently, RNA-seq was carried out for these three genotypes across the two time courses.

**Figure 3:**
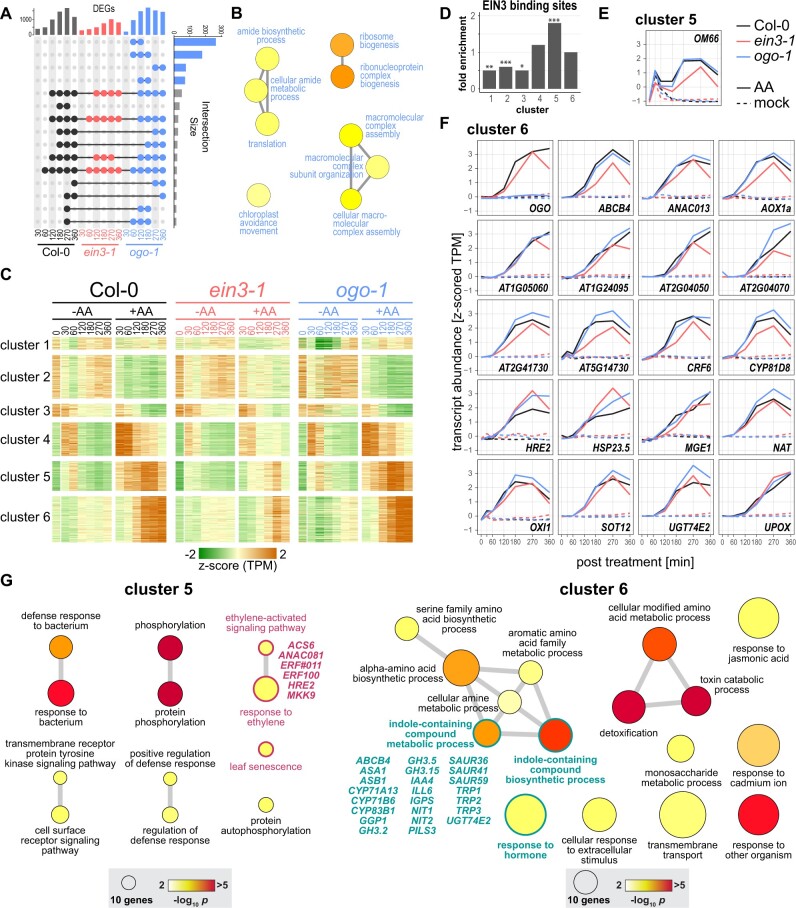
Impaired ethylene-signaling in the *ein3-1* mutant attenuates the transcriptional response to mitochondrial dysfunction. A time course RNA-seq experiment was performed by treating Col-0, *ein3-1* mutant, and *ogo-1* mutant lines with AA to induced mitochondrial stress or with water as a control treatment. Samples of three biological replicates were taken at the indicated timepoints for RNA-seq analysis. A, UpSet plot representation of overlaps in DEGs (|log2(fold change AA versus control)| > 1, FDR <0.05) for the comparisons of AA and control treatments at the same time point (Conway et al., 2017). The vertical bar chart gives the number of DEGs in the three genotypes at the different time points and the horizontal bar chart the number of overlapping DEGs in the intersects indicated by connected dots. Only the 15 largest intersects are shown. B, GO terms enriched (*P* < 0.001 after Bonferroni correction) for the *ogo-1*-specific DEGs in the four largest intersects (indicated by blue, horizontal bars) as shown in (A). C, A self-organism maps algorithm identified six clusters of DEGs with shared expression patterns across the three genotypes and time points. D, Enrichment of EIN3 binding sites in the promoters of genes in clusters 1–6. The gene lists for the six clusters were cross-referenced with a list of identified promoter binding sites for EIN3 (Chang et al., 2013). Enrichment was calculated by a hypergeometric test with asterisks indicating statistical significance (*P < 0.05, **P < 0.01, ***P < 0.001). E and F, Expression profiles in the AA and control treatments across Col-0, *ein3-1*, and *ogo-1* for the *MDS* genes (De Clercq et al., 2013). These genes were included in cluster 6 except for *OM66* in cluster 5. G, Enriched GO terms (*P* <0.001 after Bonferroni correction) for DEGs in clusters 5 and 6, respectively. Genes related to ethylene signaling in cluster 5 (magenta) or auxin signaling in cluster 6 (turquoise) are listed. Circle sizes represent the number of genes included in the GO term and circle color the significance of enrichment as indicated.

For the comparison of AA and mock treatments at the same time point, the number of differentially expressed genes (DEGs; |log2 (fold change)| >1, false discovery rate (FDR) < 0.05) was the lowest in *ein3-1*, while *ogo-1* had generally a higher number of DEGs than the wild-type, especially at early (60, 120 min) and late (360 min) time points (Figure 3A;Supplemental Data Set S1). Overlaps in DEGs across the genotypes were limited for the two earliest time points and then increased, with *ogo-1* maintaining the highest number of specific DEGs throughout the time course (Figure 3A). The four largest intersects contained only *ogo-1*-specific DEGs, and a gene ontology (GO) term enrichment analysis determined that they were related to ribosome biogenesis, translation and chloroplast avoidance movement (Figure 3B;Supplemental Data Set S2). The associated genes for the former two GO terms were largely encoding ribosomal or nucleolar proteins, while the latter (*J-DOMAIN PROTEIN REQUIRED FOR CHLOROPLAST ACCUMULATION RESPONSE 1*, *PHOTOTROPIN 1*, *PLASTID MOVEMENT IMPAIRED 1* and 2) encoded proteins that adjust the localization of chloroplasts to environmental factors (Kong and Wada, 2014). The *ein3-1* mutant shared almost all its DEGs with the other two genotypes (Figure 3A;Supplemental Data Set S1).

Based on their expression patterns under mock and AA treatments, the DEGs identified across the genotypes were separated into six clusters by a self-organizing maps clustering algorithm (Figure 3C;Supplemental Data Set S3). DEGs in clusters 2 and 3 included the DEGs with lower expression in response to AA treatment, especially in Col-0 and *ogo-1*. DEGs in cluster 4 were strongly induced early after treatment with AA in the wild-type and *ogo-1*, and these were also responsive to mock treatment and hence are touch/spray induced (Van Aken et al., 2016; Van Moerkercke et al., 2019; Xu et al., 2019). Correspondingly, several marker genes for touch responses, such as *TOUCH3*, *TOUCH4* and *WRKY DNA-BINDING PROTEIN* (*WRKY*) *40* were in the cluster 4 gene list (Supplemental Data Set S3), together with *MYC2*, a marker gene for water spray-induced responses mediated by jasmonic acid signaling (Van Moerkercke et al., 2019). For this cluster, the changes in gene expression were short-lived, with a peak already at 30–60 min, and for *ein3-1*, the transcriptional response of these genes was attenuated (Figure 3C). Although the role of ethylene and its signaling pathway in touch responses is somewhat controversial (Braam, 2005), our results and other recent work (Wu et al., 2020) suggest a function of EIN3 in controlling gene expression upon touch.

In contrast to cluster 4, DEGs in clusters 5 and 6 showed only very limited or no touch response, respectively (Figure 3C). Therefore, their considerable changes in expression are a specific response to mitochondrial dysfunction induced by AA treatment. DEGs in cluster 5 responded earlier to AA than DEGs in cluster 6, peaking at approximately 180–270 min post-treatment, after which their expression declined. The expression of genes in cluster 6 started to respond 120 min after AA treatment and continuously increased to a plateau at approximately 270 min for Col-0 and *ogo-1*. For both these clusters, the induction of DEGs was lower in *ein3-1* than in Col-0 or *ogo-1*, and also already decreased after 270 min for genes in cluster 6. Cross-referencing the gene lists for clusters 1–6 with known targets of EIN3 identified by ChIP-seq (Chang et al., 2013) showed that genes in cluster 5 were enriched (*P* < 0.001), while clusters 1–3 were depleted (*P* < 0.001–0.05), for EIN3 target genes (Figure 3D). These results suggest a role for EIN3 in the early response to AA.

Of the stress marker genes of the MDS that are controlled by the master regulator ANAC017 (De Clercq et al., 2013; Ng et al., 2013), 21 were quantifiable in the RNA-seq data, including one gene (*OM66*) in clusters 5 and 20 in cluster 6 (Figure 3, E and F). Consistent with this finding, in a transgenic line expressing a ANAC017-GFP fusion protein, fluorescence in the nucleus was first observable at approximately 0.5 h and was apparent in almost all cells at 1.5 h after AA treatment (Supplemental Figure S2). This indicates that ANAC017 was released from the ER and translocated to the nucleus, matching the observed kinetics for the upregulation of these genes. Most of these genes showed a delayed, attenuated, and/or early declining induction after AA treatment in the *ein3-1* mutant, also suggesting a role for EIN3 in the mitochondrial stress signaling pathway. Exceptions were *HSP23.5* and the ERF *HRE2*, with higher transcript levels in *ein3-1* than in the wild-type, indicating that their upregulation by AA treatment is independent of EIN3.

GO term enrichment analysis for the early responsive genes in cluster 5 revealed an enrichment for terms related to defense response, protein phosphorylation, response to ethylene, and leaf senescence (Figure 3G;Supplemental Data Set S4). Associated genes encode the ethylene biosynthesis enzyme ACS6, the ERF TFs ERF011, ERF100, and HRE2, and the MKK9. The latter is part of a signaling cascade, also involving MPK 3 and MPK6, that leads to enhanced ethylene production and signaling via phosphorylation of ACS6 and ERFs, respectively (Liu and Zhang, 2004; Meng et al., 2013). ANAC081 also positively regulates ethylene biosynthesis (Peng et al., 2022). Among the enriched GO terms for late responsive genes in cluster 6 were several related to hormone response and indole metabolism (Figure 3G;Supplemental Data Set S4).

On closer inspection of the corresponding gene list, it was apparent that many genes were related to auxin homeostasis through their involvement in auxin biosynthesis, signaling, transport, or conjugation (Casanova-Saez et al., 2021). The auxin biosynthesis-related genes encode ANTHRANILATE SYNTHASE ALPHA SUBUNIT 1 (ASA1), ANTHRANILATE SYNTHASE BETA SUBUNIT 1 (ASB1), CYTOCHROME P450 (CYP) 71B6, CYP83B1, INDOLE-3-GLYCEROL PHOSPHATE SYNTHASE (IGPS), NITRILASE 1 and 2, and TRYPTOPHAN BIOSYNTHESIS 1, 2, and 3. Genes associated with auxin-conjugation include *GRETCHEN HAGEN (GH) 3.2*, *GH3.5*, *GH3.15*, *IAA-LEUCINE RESISTANT-LIKE* (*ILL*) *6*, *CYP71A12*, *CYP71A13*, *GAMMA-GLUTAMYL PEPTIDASE 1* (*GGP1*), and the *MDS* gene *URIDINE DIPHOSPHATE GLYCOSYLTRANSFERASE* (*UGT*) *74E2*. The genes encoding auxin transporters include the *MDS* genes *ATP-BINDING CASSETTE* (*ABC*) *B4* and *PIN-LIKES* (*PILS*) *3*, while the auxin signaling-related genes included *INDOLE-3-ACETIC ACID INDUCIBLE* (*IAA*) *4* and S*MALL AUXIN UPREGULATED* (*SAUR*) *36*, *SAUR41*, and *SAUR59*.

Taken together, our approach based on a time course and three genotypes showed a sequential response to mitochondrial dysfunction that occurred in an early and a late wave of transcriptional changes. The early response suggested a primary activation of the MRR by ethylene that is dependent on EIN3, followed by a response governed by auxin-related pathways.

### A time-resolved gene-regulatory network that fine-tunes mitochondrial stress responses

**Figure 4:**
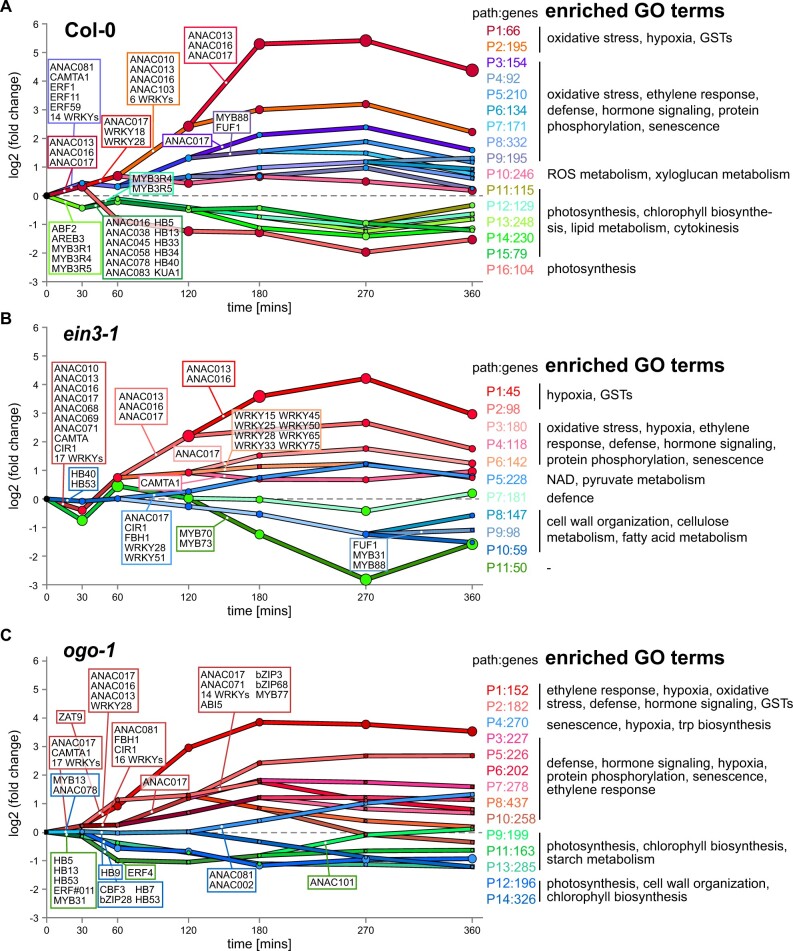
DREM analysis of the response to AA in wild-type and *ein3-1*. DREM modeling reveals differences in the sequence of regulatory events that govern the transcriptional response to mitochondrial stress induced by AA in wild-type (Col-0), *ein3-1*, and *ogo-1*. DREM models for Col-0 (A), *ein3-1* (B), and *ogo-*1 (C) show groups of co-expressed genes in 16, 11, and 14 paths, respectively, with TFs underlying the separation of genes into different paths indicated for major furcation events. Paths emanating from the three primary paths are colored in shades of red, blue, or green. The *y*-axis gives the average expression levels of genes in the paths at the indicated time points after AA treatment, and node areas are proportional to the standard deviation of the distribution of genes associated with them. Number of genes in each path and a summary of enriched GO terms (*P* <0.001 after Bonferroni correction) are indicated on the right (see Supplemental Data Sets S5–S13 for all genes, TFs associated with regulatory events and details for GO term enrichment for all paths).

To gain further insight into how the MRR to stress leads to changes in the expression of thousands of genes, and the impact a mutation of *EIN3* on this process, we performed time-resolved modeling to obtain a dynamic regulatory network of TFs and their target genes following AA treatment. For this we applied Dynamic Regulatory Events Miner (DREM) software, incorporating curated binding sites for 516 TFs from publicly available ChIP-seq and DAP-seq data sets (Schulz et al., 2012). This defines groups of genes with similar temporal expression pattern, determines time points at which the expression of genes sets diverge, and predicts TFs causal for these splits based on their binding to promoters of these genes. The final output provides groups of genes on similar temporal trajectories of expression (termed paths) and designates TFs to the underlying activation events. Using our time-resolved RNA-seq data, the DREM analysis identified 16 paths for Col-0 and 11 paths for *ein3-1* that consisted of genes with distinct biological functions (Figure 4, A and B; Supplemental Data Sets S5–S10).

The DREM model for Col-0 separated the genes into paths that arose from the three initial splits between 0 and 30 min (colored red, blue, green in Figure 4A;Supplemental Data Sets S5–S7). Paths 1 and 2 included genes most highly upregulated by AA (above eight-fold) that were regulated by ANAC013, ANAC016, and ANAC017 (TF set 2 in Supplemental Data Set S5), the three closely related ER-tethered TFs that are established regulators of mitochondrial retrograde signaling (De Clercq et al., 2013; Ng et al., 2013), validating the ability of the DREM model to predict regulatory relationships. Several stress-responsive WRKY family TFs join these ANACs in the connecting paths, including the senescence regulators WRKY25 and WRKY28 (Figure 4A; TF sets 4 and 7 in Supplemental Data Set S5; Doll et al., 2019; Tian et al., 2020). Consistent with these findings, these two paths contained all of the *MDS* genes except one and were linked to enriched GO terms related to oxidative stress and hypoxia (Figure 4A;Supplemental Data Set S7). Path 16 also originated from the same early furcation as paths 1 and 2, but the included genes were strongly downregulated (clusters 2 and 3 in Figure 3C) and associated with photosynthesis. This agrees with evidence that organelles share regulatory pathways that depend on ANACs and that they interact with RADICAL-INDUCED CELL DEATH (RCD) 1 to coordinate these responses (Meng et al., 2019; Shapiguzov et al., 2019).

From the primary furcation, paths 3–9 contain genes that are also upregulated but to a lesser degree than genes in paths 1 and 2. These genes are associated with oxidative stress, ethylene responses, hormone signaling and senescence, among others (Figure 4A;Supplemental Data Set S7). Predicted early regulators of these genes were several WRKY TFs, ANAC081, CALMODULIN-BINDING TRANSCRIPTION ACTIVATOR (CAMTA) 1, and ERF1, ERF11, and ERF59. These ERFs are involved in diverse stress-signaling pathways (Muller and Munne-Bosch, 2015), while CAMTA1 belongs to the ethylene-induced calmodulin binding protein family and is also involved in auxin signaling (Reddy et al., 2000; Galon et al., 2010). ANAC081 promotes ethylene production (Peng et al., 2022). Interestingly, ANAC017 was also a regulator of genes in path 3 resulting from a furcation event at 120 min, suggesting it does not exclusively regulate the highly induced genes in paths 1 and 2 but is also associated with hormonal and growth responses. MYB DOMAIN PROTEIN (MYB) 88 and FOREVER YOUNG FLOWER UP-REGULATING FACTOR1 (FUF1) were regulators of genes in paths 5 and 7 at the same furcation. MYB88 is a regulator of the expression of the auxin transporter genes *PINFORMED* (*PIN*) *3* and *PIN7*, and FUF1 is an ERF that suppresses senescence by activating several ethylene response DNA-binding factors (Chen et al., 2015; Wang et al., 2015).

While analyses of AA responses have largely focused on upregulated genes, in Col-0 we find several largely uncharacterized MYBs, HOMEOBOX PROTEINs (HBs), and ANACs that are predicted regulators of downregulated genes. These downregulated genes are enriched in GO terms related to cytokinesis, photosynthesis, chlorophyll biosynthesis, and lipid metabolism (Figure 4A;Supplemental Data Set S7), suggesting an active inhibition of growth and primary metabolism associated with chloroplast functions in the response to AA. In agreement with this notion, the earliest regulators of paths 11–15 predicted by the DREM model are ABSCISIC ACID-RESPONSIVE ELEMENTS-BINDING FACTOR (ABF) 2, a promoter of chlorophyll degradation (Gao et al., 2016), and MYB3R1, MYB3R4, and MYB3R5, which are positive regulators of cytokinesis (Haga et al., 2007), with the latter two maintaining a regulatory role for paths 12 and 13. Of the six ANACs initiating the split leading to paths 11, 14, and 15, four have an established function: ANAC016 and ANAC078 (together with ANAC013 and ANAC017) belong to the phylogenetically similar mitochondrial dysfunction regulators (De Clercq et al., 2013). ANAC078 also has a function in supporting protein degradation under stress conditions (Gladman et al., 2016), and ANAC045 and ANAC083 control the differentiation of sieve elements and xylem vessels, respectively (Yamaguchi et al., 2010; Furuta et al., 2014). DREM model-predicted regulators of the HB TF family for paths 11, 14, 15 are HB5, HB13, HB33, HB34, and HB40 (Figure 4A;Supplemental Data Set S5). These HBs all function in seedling development by regulating cell expansion and proliferation, which is mediated by hormonal signaling pathways (including auxin) and stress (Perotti et al., 2017). KUA1 was also a regulator of these paths, and like HBs, controls leaf cell expansion and enhances auxin accumulation (Kwon et al., 2013; Lu et al., 2014). Thus, the DREM model established a temporal sequence of regulatory events that extend our knowledge on the known TFs responsible for the induction of genes, such as ANAC017 and ANAC013, to mitigate the impact of AA on mitochondrial function. The results also identified TFs that had not been associated with mitochondrial stress, for example ERF1, ERF11, ERF59, or ANAC081, that fine-tune this response and TFs that downregulate growth and photosynthesis.

We next examined how these regulatory events changed in the *ein3-1* mutant. For *ein3-1*, the DREM model predicted only 11 paths, suggesting a less complex regulatory network than for Col-0, which fits the lower number of DEGs (Figure 4B;Supplemental Data Sets S8–S10). An obvious similarity was the conservation of ANAC013, ANAC016, and ANAC017 (i.e. the key regulators of mitochondrial stress signaling) at the earliest time point and their sustained association with the most highly upregulated genes (paths 1 and 2). Also conserved at the early time points were stress-responsive WRKYs and CAMTA1. Notably, and in contrast to Col-0 (Figure 4A), ERFs and ANAC081 were missing from these early regulators, indicative of their regulation by EIN3 (Figure 4B).

Inspection of a list of EIN3 target genes identified by ChIP-seq (Chang et al., 2013) confirmed the ethylene-dependent binding of EIN3 to the promoters of *ERF1*, *ERF11*, and *ANAC081*. In contrast to Col-0, downregulated genes were not enriched for GO terms related to photosynthesis or cytokinesis, but rather for cell wall organization and cellulose metabolism (paths 8–11; Supplemental Data Set S10). This downregulation was not driven by ANACs and HBs as in Col-0; instead, MYB31, MYB88, and FYF UPREGULATING 321 FACTOR (FUF) 1 were regulators of genes in path 9 and MYB70 and MYB73 of path 11 (Figure 4B;Supplemental Data Set S8). MYB70 and MYB73 are closely related members of the R2R3 MYB family subgroup 22 (Stracke et al., 2001). While little is known about MYB70, MYB73 and other members of this subfamily are positive regulators of auxin signaling (Zhao et al., 2014; Yang et al., 2020), and MYB31 is an interactor of phytochrome A (Zhao et al., 2014; Yan et al., 2020; Yang et al., 2020). In contrast to their regulation of upregulated genes in Col-0, MYB88 and FUF1 were predicted regulators of downregulated genes in *ein3-1* and at the later time point of 240 mins, thus indicating complexities in timing and TF hierarchies.

For *ogo-1*, the DREM model also confirmed ANAC017, ANAC013, and ANAC016 as the main regulators of the highly AA-responsive genes (Figure 4C;Supplemental Data Sets S11 and S12), together with a number of WRKY TFs. GO term enrichment analysis also determined that hypoxia-, ethylene-, and oxidative stress-related genes were prevalent in the corresponding paths (Figure 4C;Supplemental Data Set S13). Similar to Col-0, the downregulated genes were largely associated with photosynthesis and chloroplast function controlled by HB and ANAC family members. In contrast to Col-0, the primary furcation, and its derived paths, were less clearly separated over time. This suggests that regulatory networks and the coordinated expression of genes might be impaired by a loss of functional OGO.

In summary, the DREM modeling defines the chronology of regulatory events and expands our knowledge of related TFs beyond the well-known key regulators of mitochondrial dysfunction that activate the established stress response, such as ANAC017 and ANAC013. Many of these additional TFs, including EIN3, play a role in ethylene or auxin signaling and fine-tune this response to adjust growth and metabolism when mitochondrial function is restricted.

### Ethylene-activated signaling precedes auxin-related pathways in the mitochondrial stress response

Given that the attenuated AA response in *ein3-1* (Figure 3, C and F), enrichment of EIN3 binding sites (Figure 3D), ethylene-related GO term enrichment for AA-responsive DEGs (Figure 3G), and the regulatory events predicted by the DREM model (Figure 4) consistently pointed to a role for auxin and ethylene in the mitochondrial stress response, we further analyzed our RNA-seq data to gain an understanding of the transcriptional changes for the two hormonal pathways. To do this, we generated two manually curated lists that encompass genes with auxin or ethylene-related annotations in TAIR10, GO term lists, and lists from recent literature reviews involving biosynthesis, transport, conjugation, signaling, and response (Lavy and Estelle, 2016; Dubois et al., 2018; Casanova-Saez et al., 2021; Pattyn et al., 2021). We focused only on those genes that were responsive to AA treatment (Supplemental Data Sets S14 and S15).

**Figure 5:**
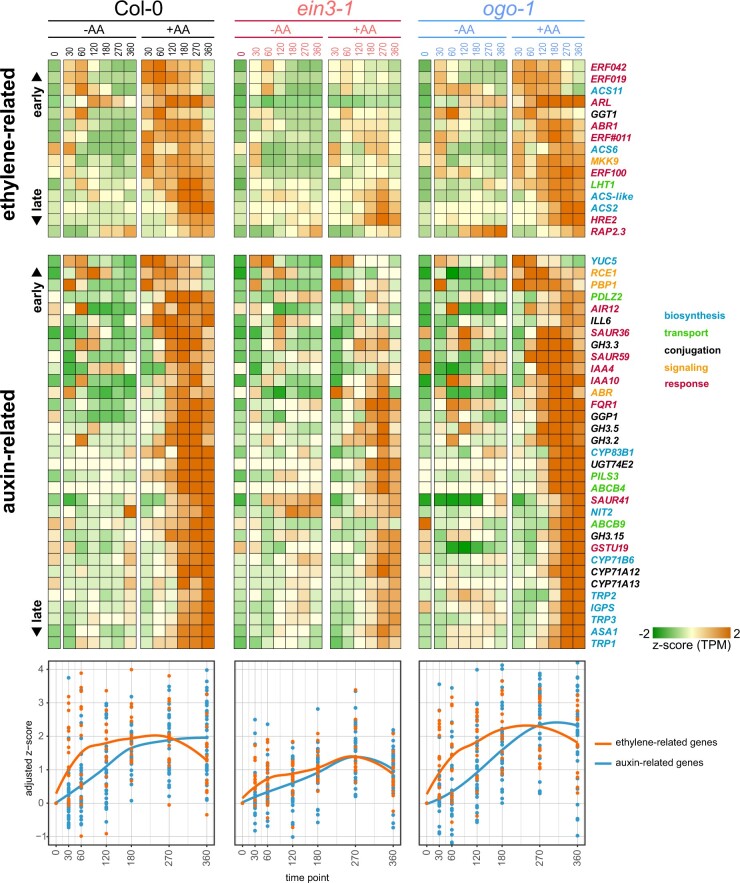
Changes in expression of genes associated with ethylene- and auxin signaling or homeostasis. From the list of DEGs responsive to AA treatment, those related to ethylene or auxin biosynthesis, transport, conjugation, signaling, or response were identified and manually curated based on their annotations in TAIR10, GO term lists, and from recent literature reviews (Supplemental Data Sets S11 and S12; Lavy and Estelle, 2016; Dubois et al., 2018; Casanova-Saez et al., 2021; Pattyn et al., 2021). A heatmap of their expression over the time course experiments is shown, with gene names colored according to their functions. Genes responding early to treatment are at the top of each heatmap, while late-responsive genes are at the bottom.

As observed for the whole DEG lists (Figure 3), the response of auxin and ethylene-related genes was attenuated in *ein3-1* with only a few exceptions (Figure 5). For the ethylene-related genes, four were early responsive to both treatments (*ACS11*, *ERF019*, *ERF042*, and the ACC conjugase gene *GAMMA-GLUTAMYL TRANSPEPTIDASE* (*GGT*) *1*) but showed a more pronounced induction after spraying with AA. Three more ERFs were among the earliest AA-induced ethylene-related genes, namely *ERF#011*, *ERF100*, and *ABR1*, indicating an early activation of the ethylene signaling pathway after AA treatment. Also, early upregulated was the *ARGOS-LIKE* (*ARL*) gene, which encodes a member of the ARGOS family of proteins. *ARL* expression is induced by ethylene in an EIN3-dependent manner, as confirmed by our RNA-seq data, and controls organ size (Hu et al., 2006; Rai et al., 2015). *RAP2.3* had a late and similar response to both treatments in Col-0, but a stronger response to mock spray in *ogo-1* (Figure 5). *RAP2.3* belongs to the class VII ERFs, which are part of the N-end rule pathway and regulators of hypoxia responses (Hartman et al., 2019). *HRE2*, another class VII ERF that is a close neighbor to AOX1A in the co-expression network (Figure 1A), was also upregulated later in the time course at 120 min and remained highly expressed. *MKK9* and *ACS6* were already upregulated at 30 min and throughout the whole time course, while *ACS2* was strongly upregulated (>16-fold) beyond 270 min after AA treatment.

Among the earliest auxin-related genes upregulated by AA treatment was *YUCCA5* (*YUC5*; Figure 5). This gene encodes one of eleven isoforms of the enzyme that catalyzes the final step in the main auxin biosynthesis pathway (Casanova-Saez et al., 2021), and more specifically in roots and young vegetative tissues (Woodward et al., 2005). There is also evidence that YUC5, together with other YUC family members, is localized to the ER membrane (Kriechbaumer et al., 2016). Also early induced is *RUB1 CONJUGATING ENZYME 1* (*RCE1*), encoding a member of the SCF^TIR^ complex. This complex targets AUX/IAA proteins for degradation, leading to the subsequent activation of auxin-induced genes. RCE1 also plays a role in regulating ethylene biosynthesis, as its mutation leads to ethylene overproduction (Larsen and Cancel, 2004). Many of the auxin genes that responded at 120 min after AA treatment were related to the auxin response, that is *IAA4*, *IAA10*, *SAUR36*, and *SAUR59* (Figure 5). The two *IAA* genes encode transcriptional repressors of auxin signaling (Lavy and Estelle, 2016), and their upregulation might block the repressive impact of auxin on mitochondrial signaling (Ivanova et al., 2014; Kerchev et al., 2014). Both *SAUR* genes have been implicated in the co-ordination of auxin and brassinosteroid signaling in development (Yu et al., 2011), with SAUR36 also promoting leaf senescence (Hou et al., 2013).

Another set of genes showing an AA response at this time point is related to auxin conjugation, that is *GH3.2*, *GH3.3*, *GGP1*, *ILL6*, *UGT74E2* (Figure 5). These genes encode enzymes that modulate the concentration of IAA (the free active form of auxin) through the (reversible) inactivation of IAA via conjugation to low molecular weight metabolites such as sugars or amino acids (Casanova-Saez et al., 2021). The genes induced by AA after 180 min or later included several auxin transporter genes (*ABCB4*, *ABCB9*, *PILS3*) and genes encoding enzymes involved in IAA precursor biosynthesis (*TRYPTOPHAN BIOSYNTHESIS* (*TRP*) *1*, *TRP2*, *TRP3*; *ANTHRANILATE SYNTHASE ALPHA SUBUNIT* (*ASA*) *1*; *INDOLE-3-GLYCEROL PHOSPHATE SYNTHASE*; *CYP71B6*; *NITRILASE 2*; Figure 5). The upregulation of these genes coincides with the downregulation of many AA-stress marker genes (Figure 3F) and suggests that increased auxin biosynthesis and altered auxin distribution inhibit the mitochondrial stress response, as observed after external auxin application (Ivanova et al., 2014; Kerchev et al., 2014).

Taken together, these results reveal that the complex interplay of components of the ethylene and auxin signaling pathways modulates mitochondrial retrograde signaling. The early upregulation of ethylene marker genes, activation of the MKK9–MPK3/6 module, and subsequent activation of ethylene biosynthesis genes suggest an initiation of ethylene signaling pathways that precedes the activation of auxin. The induction of auxin biosynthesis by ethylene is well documented (Stepanova et al., 2005; Stepanova et al., 2008) and might represent an important feedback mechanism in the regulation of mitochondrial retrograde signaling.

### ChIP-seq identifies auxin- and ethylene-related genes targeted by ANAC017

**Figure 6:**
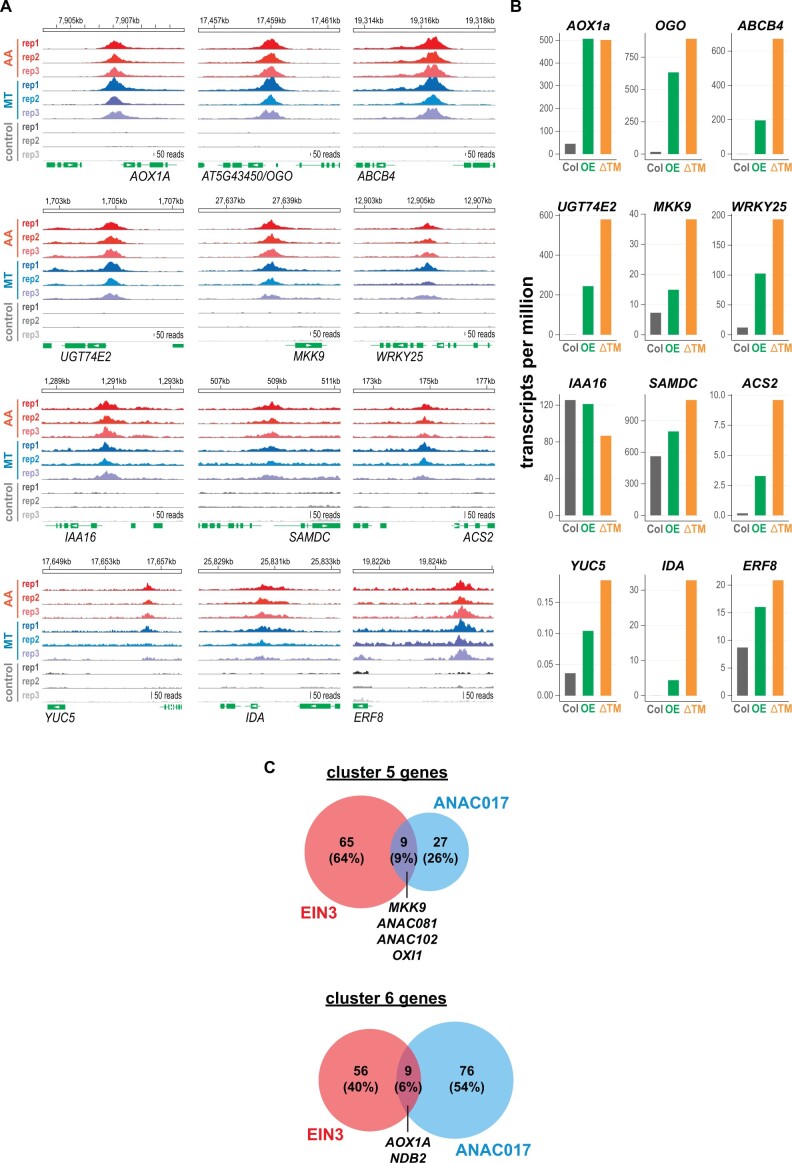
ANAC017 binds to the promoters of several ethylene- and auxin-related genes. A, Binding of ANAC017 to the promoters of target genes was determined by ChIP-seq experiments using a transgenic line expressing a GFP-ANAC017 fusion protein under the control of the native *ANAC017* promoter. ChIP-seq was performed after induction of mitochondrial stress by spraying with AA or MT, or with water as a control treatment. Using MACS2 software for peak detection (Gaspar, 2018), the 200 most significant target genes for the AA treatment, which had an enrichment factor of at least above 7 and a −log10(q) above 42, were determined (Supplemental Data Set S13). Of these genes, 178 were also highly significantly enriched (−log10(q) <5) under MT treatment (Supplemental Data Set S13). Shown are read coverages around the promoters of associated genes for three biological replicates. *AOX1A* is also given for comparison as a known ANAC017 target (De Clercq et al., 2013). Green boxes indicate the full-length cDNA location and orientation at gene loci. B. Expression levels of ANAC017 target genes in two *ANAC017* overexpression lines (*ANAC017OE3, OE; ANAC017ΔTMOE3, ΔTM*). Data were retrieved from a previously published experiment (Meng et al., 2019). C. Regulation of mitochondrial stress-responsive genes by ANAC017 and EIN3. The Venn diagram shows the overlap in the AA-responsive genes in clusters 5 and 6 (Figure 3C) whose promoters bind ANAC017 or EIN3. The target gene list of EIN3 was retrieved from a previously published ChIP-seq experiment (Chang et al., 2013). Relevant genes regulated by both TFs are indicated.

The involvement of ethylene and auxin signaling pathways in modulating the MRR raised the question of how these signaling pathways are activated upon mitochondrial dysfunction. A prime target for further investigation was ANAC017 given that it is a key regulator of mitochondrial stress signaling. We therefore conducted ChIP-seq experiments by treating Arabidopsis seedlings expressing a GFP-ANAC017 fusion protein with AA and MT to induce a mitochondrial stress response. For the controls, we used seedlings sprayed with 0.1% Tween solutions. We sampled the seedlings at 180 min after treatment. Among the 200 most highly enriched target genes were the *MDS* genes *AOX1A* and *OGO*, confirming their regulation by ANAC017 and thus validating the ChIP-seq experiment (Figure 6A;Supplemental Data Set S16; De Clercq et al., 2013). Highly enriched ChIP-seq peaks were detected for four auxin-related genes: the auxin transporter gene *ABCB4*, the gene encoding the auxin-conjugating enzyme UGT74E2, the transcriptional repressor gene *IAA16*, and the auxin biosynthesis gene *YUC5* (Figure 6A;Supplemental Data Set S16). These genes were also induced by AA in the time course experiment (Figure 5).

ANAC017 also binds to the promoters of the five ethylene-related genes with functions in signaling (*MKK9*, *ERF8*, *INFLORESCENCE DEFICIENT IN ABSCISSION* (*IDA*)) and biosynthesis (*ACS2*, *S-ADENOSYLMETHIONINE DECARBOXYLASE* (*SAMDC*)). Their regulation by ANAC017 is further supported by the higher expression of these genes, except for *IAA16*, in plants overexpressing *ANAC017* (Figure 6B;Meng et al., 2019) and their attenuated induction in an *anac017* mutant line (Supplemental Figure S3), the latter also confirmed by earlier reports (Ng et al., 2013). Several AA-responsive genes regulated by ANAC017 also have EIN3 binding sites, as identified by Chang et al. (2013), indicating their dual regulation by both TFs (Figure 6C). Thus, ANAC017 not only activates genes directly involved in the stress response, such as *AOX1A*, but also induces the expression of genes of the ethylene (in combination with EIN3) and auxin signaling pathways.

### The MKK9–MPK3/6 signaling pathway is part of the retrograde response

**Figure 7:**
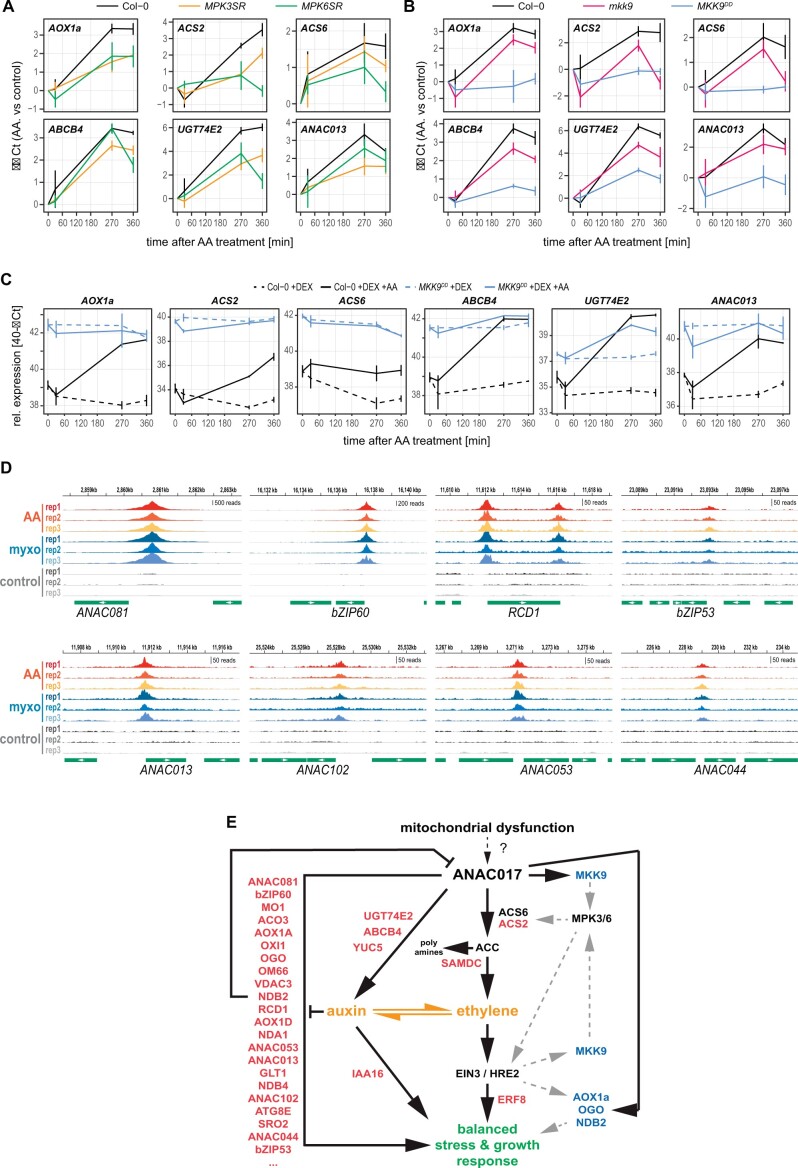
Co-ordination of retrograde signaling and growth by ANAC017, MKK9, and MPK3/6. MPK3, MPK6, and MKK9 are regulators of the mitochondrial dysfunction response (A, B, and C). The induction of genes after AA treatment is attenuated in *mpk3*, *mpk6* (A), and *mkk9* (B) mutant lines, while the apparent diminished response of these genes in the *MKK9^DD^* line (B) is based on their already elevated expression due to induction by DEX, confirming a MKK9-dependent induction of these genes (C). Seedlings were pretreated with NA-PP1 to deactivate MPK3/MPK6 function (A) or DEX to induce *MKK9^DD^* expression (B and C) 12 h before subsequent treatment with AA or water. Samples were then taken at the indicated timepoints for RNA extraction and RT–qPCR. Given are the means of the ΔΔCt ± se values (AA versus control) (B) and relative transcript abundance expressed as means of 40−ΔCt ± se values (C) of three biological replicates. D, ANAC017 directly binds to the promoters of TFs involved in the regulation of growth, senescence and stress responses. Shown are genome browser representations of ChIP-seq reads derived from a transgenic line expressing a GFP-ANAC017 fusion protein after treatment with AA, MT and water (control). Green bars indicate the position and orientation of the full-length cDNA. E, Model for the integration of retrograde signaling with plant growth by the direct action of ANAC017, an MKK9–MPK3/6 signaling cascade, and the auxin/ethylene interaction. While ANAC017 directly induces the expression of stress genes such as *AOX1A*, *NDB2*, or *OGO*, it also activates in parallel components of the ethylene (MKK9, ACS2, ERF8, SAMDC) and auxin (*YUC5*, *UGT74E2*, *ABCB4*, *IAA16*) pathways. EIN3 also targets *AOX1A*, *NDB2*, and *MKK9*, allowing for dual regulation by the two TFs, while HRE2 binds to the promoters of *AOX1A* and *OGO*. In addition, the targeting of other TF genes such as *ANAC081*, *bZIP60*, and *RCD1* by ANAC017 allows for the activation of further transcriptional cascades to fine-tune and balance the acute stress response with plant growth and also a negative feedback loop via RCD1. Genes associated with steps in the pathway and directly targeted by ANAC017 are highlighted in red (ordered by the statistical significance; Supplemental Data Set S13), while genes dually targeted by ANAC017 and EIN3 or HRE2 are highlighted in blue. Reciprocal interaction of ethylene and auxin is indicated by orange arrows. See text for details.

Current evidence suggests that stress-induced activation of the MKK9–MPK3/6 module leads to the phosphorylation of ACS2 and ACS6. The phosphorylation of these two ethylene biosynthesis enzymes results in their stabilization and subsequent enhanced ethylene production (Liu and Zhang, 2004; Zhao and Guo, 2011). The upregulation of *MKK9* after AA treatment (Figures 3 and 5) and the binding of ANAC017 to the *MKK9* promoter (Figure 6) suggested a role for MKK9 and/or MPK3/6 in the mitochondrial signaling pathway. To test this notion, we used the *mkk9* mutant, a transgenic line carrying a constitutively active and dexamethasone (DEX)-inducible MKK9 allele (*MKK9^DD^*), as well as the *MPK3SR* and *MPK6SR* transgenic lines, which are chemically inducible *mpk3 mpk6* double mutants in the presence of 4-amino-1-tert-butyl-3-(10-naphthyl) pyrazolo [3,4-d] pyrimidine (NA-PP1; Xu et al., 2008, 2016). For this, the *mkk9*/*MKK9^DD^* and *MKP3SR*/*MPK6SR* lines, together with the wild type as a control, were treated with DEX and NA-PP1, respectively, 12 h before the subsequent application of AA (Figure 7, A and B). The AA induction of the *MDS* genes *AOX1A*, *ABCB4*, *UGT74E2*, and *ANAC013* as well as the ethylene biosynthesis genes *ACS2* and *ACS6* was attenuated in the lines lacking functional MKK9, MPK3, or MPK6 (Figure 7, A and B). An almost complete loss of an AA response was detected for these genes in the *MKK9^DD^* line (Figure 7B). This was the result of their already induced expression by the elevated *MKK9^DD^* levels after DEX pretreatment, which did not further change after subsequent AA treatment (Figure 7C). Together, these results demonstrate that MKK9, MPK3, and MPK6 are involved in regulating mitochondrial stress response genes such as *AOX1A*.

### ANAC017 integrates mitochondrial signaling, growth, and stress responses

The list of ANAC017 targets identified by ChIP-seq also contains genes encoding TFs involved in regulating wider stress responses and plant growth (Figure 7D, Supplemental Data Set S16). These include genes encoding the ANAC TF family members ANAC081, ANAC013, ANAC102, ANAC053, and ANAC044, which control a wide range of growth processes and stress responses, including the cell cycle, senescence, responses to photooxidative damage, and seed germination (Christianson et al., 2009; Takasaki et al., 2015; D'Alessandro et al., 2018; Takahashi et al., 2019; Nagahage et al., 2020), with ANAC013 and ANAC053 also directly involved in the mitochondrial dysfunction response (De Clercq et al., 2013). The most significantly enriched region was located in the promoter of *ANAC081*, which promotes ethylene biosynthesis (Supplemental Data Set S16; Peng et al., 2022). bZIP60 and bZIP53 are key regulators of the unfolded protein response of the endoplasmic reticulum (UPR^ER^) and metabolic reprogramming under low energy stress, respectively (Iwata et al., 2008; Dietrich et al., 2011). Increased mitochondrial respiration was recently shown to protect the ER from reductive stress (Fuchs et al., 2022); the activation of these two bZIPs by ANAC017 provides a direct mechanism for functional cross-talk between the two organelles. In addition, *RCD1* is also a direct target of ANAC017 to allow for the reciprocal regulation of these two TFs, as the RCD1 protein also binds to the ANAC17 protein to act as a negative regulator of its activity (Shapiguzov et al., 2019). Thus, ANAC017 integrates the primary response to mitochondrial dysfunction with plant growth and controls its own activity via a negative RCD1-dependent feedback loop.

## Discussion

The data presented in this study provide evidence for the presence of distinct signaling pathways emanating from the master regulator ANAC017 (Figure 7E). One is the known direct activation of mitochondrial stress-mitigating genes, such as *AOX1A*, *OM66*, *VDAC3* or more recently *ACONITASE* 3, for a fast response that alleviates the over-reduction of the mitochondrial electron transport chain and allows for metabolic acclimation (De Clercq et al., 2013; Ng et al., 2013; Meng et al., 2019; Pascual et al., 2021).

While a reciprocal antagonistic relationship between the MRR and auxin has been established (Ivanova et al., 2014; Kerchev et al., 2014), our study provides a mechanism through the activation of auxin-related genes by ANAC017 as well as ethylene pathway-related genes (Figure 7E). The interaction of auxin and ethylene signaling pathways is complex, with synergistic and antagonistic relationships, depending on the analyzed processes and tissues (Muday et al., 2012). Reciprocal regulation of both biosynthetic pathways has been demonstrated, with increased levels of auxin leading to the induction of ethylene biosynthesis (Ruzicka et al., 2007; Stepanova et al., 2007; Swarup et al., 2007), and ethylene also promoting auxin biosynthesis (Stepanova et al., 2005, 2008). This is further complicated by the localized synthesis of both phytohormones and their transport between tissues (Brumos et al., 2018). Our findings suggest that ANAC017 balances the timing and amplitude of the MRR via both phytohormones. This agrees with recent studies showing an involvement of ethylene in the stimulation of the MRR (Wang and Auwerx, 2017; Jurdak et al., 2021). In contrast, auxin represses the MRR and thus provides a regulatory feedback loop (Ivanova et al., 2014; Kerchev et al., 2014). The diminished AA response in the *ein3-1* mutant demonstrates that ethylene promotes the MRR and that the induction of many genes in the downstream cascade is dependent on EIN3. Importantly, components of both phytohormone pathways are under direct control of ANAC017, and the two plant hormones are also major regulators of plant development (Muday et al., 2012), thus providing a mechanism to control mitochondrial stress signaling and growth via the action of the same TF (Figure 7E).

The early upregulation of ethylene pathway genes and the binding of ANAC017 to the promoters of some of these genes, especially *MKK9* and *ACS2*, suggests that an increase in ethylene biosynthesis promotes the early mitochondrial stress response. MKK9 also stimulates the MPK3/6 cascade, leading to the phosphorylation and stabilization of another ethylene biosynthesis enzyme, ACS6 (Liu and Zhang, 2004; Xu et al., 2008). Consistent with this observation, the activation of MPK3 and MPK6 after oxygen deprivation, AA, or cyanide treatment occurs within 2 h (Chang et al., 2012), that is in parallel with the upregulation of genes in our RNA-seq experiment and the accumulation of ANAC017 in the nucleus. Our work suggests that MKK9 functions as an activating kinase following induction by ANAC017. A role of ethylene and EIN3, the master regulator of ethylene signaling, in the MRR is also supported by the dual control of *AOX1A*, *MKK9*, and *NDB2* by both ANAC017 and EIN3 (Figure 7). In addition, the auxin efflux carriers PIN1 and PIN6 are also targeted by MPK3/6 (Ditengou et al., 2018; Dory et al., 2018). This may explain the identification of PIN1 as one of the regulators of *AOX1A* expression (Ivanova et al., 2014). Hence, the upregulation of *MKK9*, and the downstream activation of the MPK3/6 cascade, by ANAC017 potentially has a broader effect on growth (Figure 7E). In agreement with this notion, increased expression of *MKK9* has a similar effect as *ANAC017* overexpression in promoting senescence (Xu et al., 2008; Zhou et al., 2009; Meng et al., 2019; Broda et al., 2021). Also, the involvement of MPK3/6 in the MRR provides a direct link to methylerythritol cyclodiphosphate-mediated chloroplast retrograde signaling, which also results in a reduction in auxin levels (Zeng et al., 2022).

In summary, our findings establish a model for the roles of ethylene and auxin in controlling the MRR via direct activation by ANAC017 (Figure 7E). According to this model, early activation of key genes involved in ethylene signaling and biosynthesis, including *EIN3*, *ANAC081/ATAF2*, and *MKK9*, promotes the MRR to mitigate mitochondrial dysfunction. Subsequent activation of auxin biosynthesis, transport, and conjugation, also enhanced by ethylene-induced auxin biosynthesis, increases IAA levels, which represses the MRR. This feedback loop, in conjunction with another antagonistic feedback loop involving RCD1 (Shapiguzov et al., 2019), allows for a finely tuned response to mitochondrial dysfunction. Additionally, it provides a mechanism for the coordination with growth and ER-stress via ANAC017-regulated TFs such as bZIP60.

## Materials and methods

### Plant material and growth conditions

The *A.thaliana* Columbia-0 (Col-0; CS70000) accession was used as the wild-type control for all experiments. The *ein3-1*, *rao2-1*, and *proANAC017:GFP-ANAC017* lines were described previously (Binder et al., 2007; Ng et al., 2013; Meng et al., 2022). The *ogo-1* mutant was obtained from the Nottingham Arabidopsis Stock Center (SALK_107806). T-DNA insertion in the gene *AT5G43450* (*OGO*) was confirmed by PCR and Sanger sequencing. Additionally, the RNA-seq data confirmed T-DNA integration and gene knockout without affecting the expression of neighboring genes (Supplemental Figure S1). GUS reporter lines for *OGO* (*proOGO*-*GUS*) were generated by polymerase chain reaction (PCR) amplification of the 1-kb region upstream of the *OGO* translation start site and cloning into the vector pGPTV-BAR using Gibson assembly (Gibson et al., 2009). The 35S:GFP-ANAC017 line was generated by PCR amplification of the ANAC017 coding region and subsequent assembly into the vector pK7WGF2 using Gateway methodology (Invitrogen). All constructs were verified by Sanger sequencing. Transgenic lines carrying these constructs were generated by *Agrobacterium tumefaciens*-mediated transformation using the floral dip method (Clough and Bent, 1998). Representative lines were selected from progeny of 30 independent events. Chemically inducible *mpk3 mpk6* double mutants (*MPK3SR*, genotype *mpk3 mpk6 proMPK3:MPK3^TG^*; *MPK6SR*, genotype: *mpk3 mpk6 proMPK6:MPK6^YG^*), the *mkk9* mutant and the transgenic line carrying a constitutively active *MKK9* allele (*MKK9^DD^*) were described previously (Xu et al., 2008, 2014).

For experiments on plates, seeds were surface sterilized, stratified at 4°C for 48 h, and sown on B5 medium supplemented with 1% sucrose and 0.75% (w/v) agar. Plants were then grown in a 14-h/10-h light/dark photoperiod at 22°C and 100 μmol m^−2^ s^−1^ photosynthetic photon flux density supplied by fluorescent tubes (4000 k cool white). For the AA time course RNA-seq experiment, seedlings were grown in constant light (100 μmol m^−2^ s^−1^) at 22°C. For AA treatments, plants grown on plates for 10 days were sprayed with either 50-µM AA/0.01% Tween-20 solution or only 0.01% Tween solution for the controls. 4-amino-1-tert-butyl-3-(1′ naphthyl) pyrazolo [3,4-d] pyrimidine (NA-PP1, 2 μM) or DEX (15 µM) was applied 12 h before AA treatment as indicated. Plants were harvested at the indicated time points in parallel for both treatments. For senescence experiments and submergence treatments, plants were grown in soil in controlled environment rooms under a 14-h/10-h light/dark photoperiod at 22°C and 120 μmol m^−2^ s^−1^ photosynthetic photon flux density. Submergence experiments were performed as described previously (Meng et al., 2020).

### Biochemical assays

β-glucuronidase (GUS) reporter assays were performed as described previously (Bowling et al., 1994). For chlorophyll extraction, leaf material (50 mg) was incubated in pre-chilled 100% methanol overnight in the dark. After complete extraction, chlorophyll in the supernatants was quantified at 666 nm and 653 nm with a spectrophotometer (BMG, ClarioSTAR) as previously described (Lichtenthaler, 1987). The maximum quantum yield of photosystem II (Fv/Fm) was determined after 20 min of dark acclimation using a Chlorophyll Fluorescence System (Photon Systems Instruments, Czech Republic).

### Confocal microscopy

Fluorescence imaging was performed with a Zeiss LSM780 confocal microscope using an LD C-Apochromat 40×/1.1 water-immersion objective. GFP fluorescence was excited at 488 nm using an argon ion laser and subsequently detected at 490–600 nm.

### RT–qPCR

Total RNA was isolated from homogenized tissue using the Spectrum Plant Total RNA kit (Sigma) according to the manufacturer’s protocol. Removal of genomic DNA was performed with an On-Column DNase I (Sigma) digestion kit prior to RNA elution. For RT–qPCR, cDNA was generated using a Tetro cDNA Synthesis Kit (Bioline) and then used to perform qPCR with a SensiFAST SYBR & Fluorescein Kit (Bioline) on a QuantStudio 12K Flex Real-Time PCR system (Applied Biosystems). The PCR conditions were 95°C for 2 min; 40 cycles of 95°C for 20 sec; 60°C for 30 s with primers listed in Supplemental Data Set S17. Data were analyzed using QuantStudio 12K Flex software (Applied Biosystems). All experiments were performed with at least three biological replicates consisting of three to four pooled seedlings from independent plates.

### RNA-seq and bioinformatic analysis

Total RNA was extracted as described above for three biological replicates (three to four pooled seedlings from independent plates per replicate) for each genotype and sampling time point. RNA-seq libraries were generated with a TruSeq Stranded mRNA Library Prep Kit and sequenced on a NextSeq500 instrument (both Illumina) as 70-bp reads with an average quality score (Q30) of above 93% and an average of 13.2 million reads per sample. Quality control was performed using FastQC software (https://www.bioinformatics.babraham.ac.uk/projects/fastqc/).

Transcript abundances as transcripts per million and estimated counts were quantified on a gene level by pseudo-aligning reads against a k-mer index build from the representative transcript models downloaded for the Araport 11 annotation (Cheng et al., 2017) using a k-mer length of 31 using the kallisto program with 100 bootstraps (Bray et al., 2016). The program sleuth with a likelihood ratio test was used to test for differential gene expression (Pimentel et al., 2017). Only genes with at least five counts in half of all samples per time point were included in this analysis. DEGs were called with a false discovery rate FDR < 0.05 and a |log2 (fold change)| > 1. Overlaps in the list of DEGs across the different genotypes were identified and represented using UpSet plots (Conway et al., 2017). Further analyses, hierarchical clustering, and generation of heat maps were performed using the Partek Genomics software suite version 6.16 (Partek Incorporated, http://www.partek.com/). GO term enrichment analysis was performed using the ClueGO plugin for Cytoscape (Bindea et al., 2009).

DREM analysis was performed as described with default parameters (Schulz et al., 2012). The input file contained TF-gene interactions for 516 TFs curated from published ChIP-seq and DAP-seq studies (O'Malley et al., 2016; Narsai et al., 2017; Zander et al., 2020) and confirmed interactions obtained from the Arabidopsis Gene Regulatory Information Server (Yilmaz et al., 2011).

### ChIP-seq

The *proANAC017*:*GFP-ANAC017* line was grown under a 16-h day/8-h night photoperiod at 23°C with 100 μmol·m^−2^s^−1^ photosynthetic photon flux density for 12 days on B5 medium with 1% sucrose, 0.8% (w/v) agar. Seedlings were sprayed with 50-µM AA/0.01% Tween-20 solution, 50-µM MT/0.01% Tween20 solution, or 0.01% Tween-20 for the control treatment. The ChIP experiments were performed as described previously (Zander et al., 2020) on three biological replicates (three to four pooled seedlings from independent plates per replicate) per treatment with seedlings harvested 3-h post-treatment for chromatin purification. Ten µg of anti-GFP antibody (Invitrogen Cat# A11122) was coupled to 50-µL Dynal Protein A Dynabeads (Thermo Fisher Scientific) overnight at 4°C and equal amounts of sonicated chromatin subsequently added for overnight incubation at 4°C. A mock sample without antibody input was used as a control. The beads were washed twice with low salt buffer (50-mM Tris–HCl, pH 7.4, 150-mM NaCl, 2-mM EDTA, 0.5% Triton X-100), high salt buffer (50-mM Tris–HCl, pH 7.4, 500-mM NaCl, 2-mM EDTA, 0.5% Triton X-100), and final wash buffer (50-mM Tris–HCl, pH 7.4, 50-mM NaCl, 2-mM EDTA). Samples were de-crosslinked and digested with proteinase K before the DNA was purified using a QIAquick PCR Purification Kit (Qiagen).

ChIP-seq libraries were generated with an Accel-NGS 2S Plus DNA Library Kit following the manufacturer’s instructions (Swift Biosciences) and sequenced on a NextSeq500 platform (Illumina) with an 84-bp read length. Reads were mapped to the Arabidopsis reference genome (TAIR10) using Bowtie2 (Langmead and Salzberg, 2012). ChIP-seq peaks were called with MACS2 software using default parameters (Zhang et al., 2008).

### Statistical analyses

The statistical analyses were performed by one-way analysis of variance (ANOVA). The ANOVA tables are provided in Supplemental Data Set S18.

### Accession numbers

Sequence data from this article and further gene information can be found in the TAIR10 database (www.ararbidopsis.org) and Supplemental Data Set S1 using the gene names and AGI identifiers given in this article. RNA-seq read data and ChIP-seq read data were deposited at the NCBI SRA database under project ID PRJNA745499 and PRJNA699617, respectively.

## Supplemental data

The following materials are available in the online version of this article.

**Supplemental Figure S1.** Characterization of the *ogo-1* mutant.

**Supplemental Figure S2.** Accumulation of ANAC017-GFP fusion protein in the nucleus after AA treatment.

**Supplemental Figure S3.** Induction of ANAC017 target genes is attenuated in an *anac017* mutant line.

**Supplemental Data Set S1.** DEGs for the comparison of AA versus mock treatments.

**Supplemental Data Set S2**. GO term enrichment (*P* < 0.001) for OGO-specific DEGs shown in Figure 3B.

**Supplemental Data Set S3.** z-scored TPM values for DEGs (Supplemental Data Set S1) after clustering using a self-organizing maps algorithm (Figure 3C).

**Supplemental Data Set S4.** GO term enrichment (*P* < 0.001) for gene clusters in Figure 3, C and G.

**Supplemental Data Set S5.** Output of the DREM model for Col-0 (Figure 4A).

**Supplemental Data Set S6.** Genes in each path for the DREM model of Col-0 (Figure 4A).

**Supplemental Data Set S7.** GO term enrichment (*P* < 0.001) for genes in Col-0 DREM model paths (Figure 4A).

**Supplemental Data Set S8.** Output of the DREM model for *ein3-1* (Figure 4B).

**Supplemental Data Set S9.** Genes in each path for the DREM model of *ein3-1* (Figure 4B).

**Supplemental Data Set S10.** GO term enrichment (*P* < 0.001) for genes in *ein3-1* DREM model paths (Figure 4B).

**Supplemental Data Set S11.** Output of the DREM model for *ogo-1* (Figure 4C).

**Supplemental Data Set S12.** Genes in each path for the DREM model of *ogo-1* (Figure 4C).

**Supplemental Data Set S13.** GO term enrichment (*P* < 0.001) for genes in *ogo-1* DREM model paths (Figure 4C).

**Supplemental Data Set S14.** Ethylene-related DEGs responsive to AA treatment (Figure 5).

**Supplemental Data Set S15**. Auxin-related DEGs responsive to AA treatment (Figure 5).

**Supplemental Data Set S16.** ChIP-seq peaks for the 200 most statistically significant target genes of ANAC017.

**Supplemental Data Set S17.** Primers used in this study.

**Supplemental Data Set S18.** ANOVA tables for statistical analyses.

## Supplementary Material

koac177_Supplementary_Data

## Notes

O.B., C.H., and J.W. designed the research. C.H., L.C.L., L.Y., and O.B. performed the research. C.H., L.C.L., L.Y., O.B., M.G.L., and J.W. collected, analyzed or interpreted the data; O.B., C.H., and J.W. wrote the article with contributions by all the authors.

The author responsible for distribution of materials integral to the findings presented in this article in accordance with the policy described in the Instructions for Authors (https://academic.oup.com/plcell) is: James Whelan (j.whelan@latrobe.edu.au).
