# Supplementary material for: The retrograde signaling regulator ANAC017 recruits the MKK9–MPK3/6, ethylene, and auxin signaling pathways to balance mitochondrial dysfunction with growth
Source: Plant Cell. 2022 Jun 16;34(9):3460–81. doi: 10.1093/plcell/koac177 (PMC9421482; doi:10.1093/plcell/koac177)
Supplement: koac177_Supplementary_Data [file koac177_supplementary_data.zip › tpc.22.00293_SupplementalFigures.pdf]

Supplemental Data. He et al. (2022). The retrograde signaling regulator ANAC017 recruits the MKK9-MPK3/6, ethylene, and auxin signaling pathways to balance mitochondrial dysfunction with growth. Plant Cell.

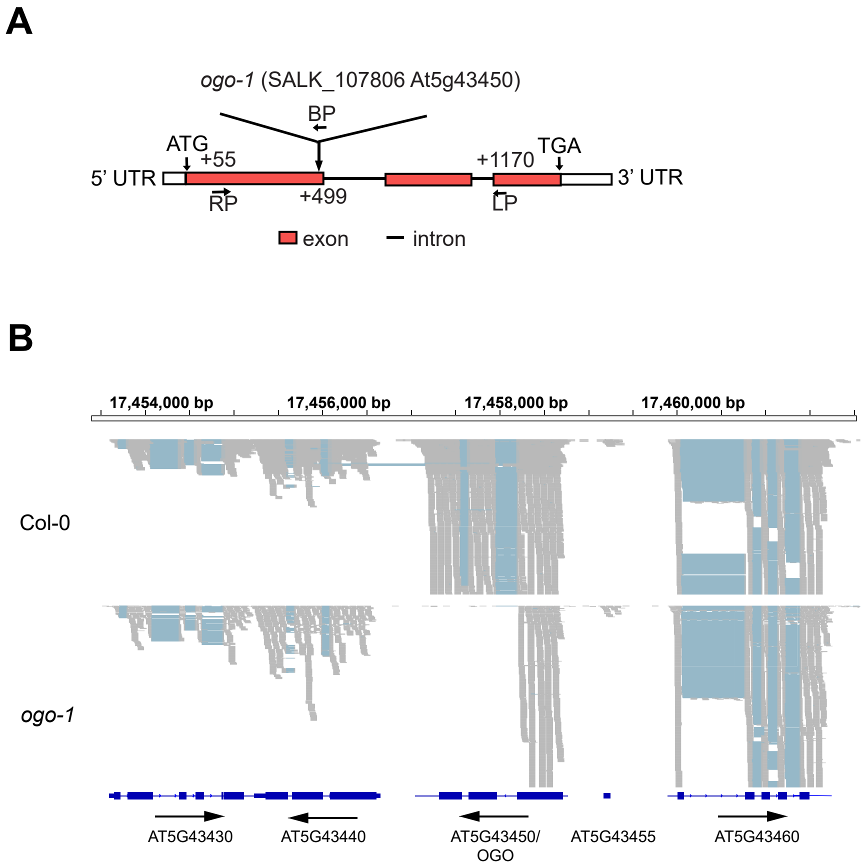

Supplemental Figure 1: Characterization of the *ogo-1* mutant (supports Figures 2, 3, 4, and 5).

A. Schematic representation of T-DNA insertion site in the *ogo-1* mutant (SALK_107806). Primer binding site used for the characterization of the insertion site are indicated. PCR and DNA sequencing confirmed the integration of the T-DNA at the end of exon 1.

B. Genome browser view for read alignments of RNA-seq data for antimycin A treated plants. No reads could be detected beyond the T-DNA insertion site in exon 1, confirming the non-functionality of the *OGO* gene in the *ogo-1* mutant. Also, expression of neighboring genes was not affected by the T-DNA integration.

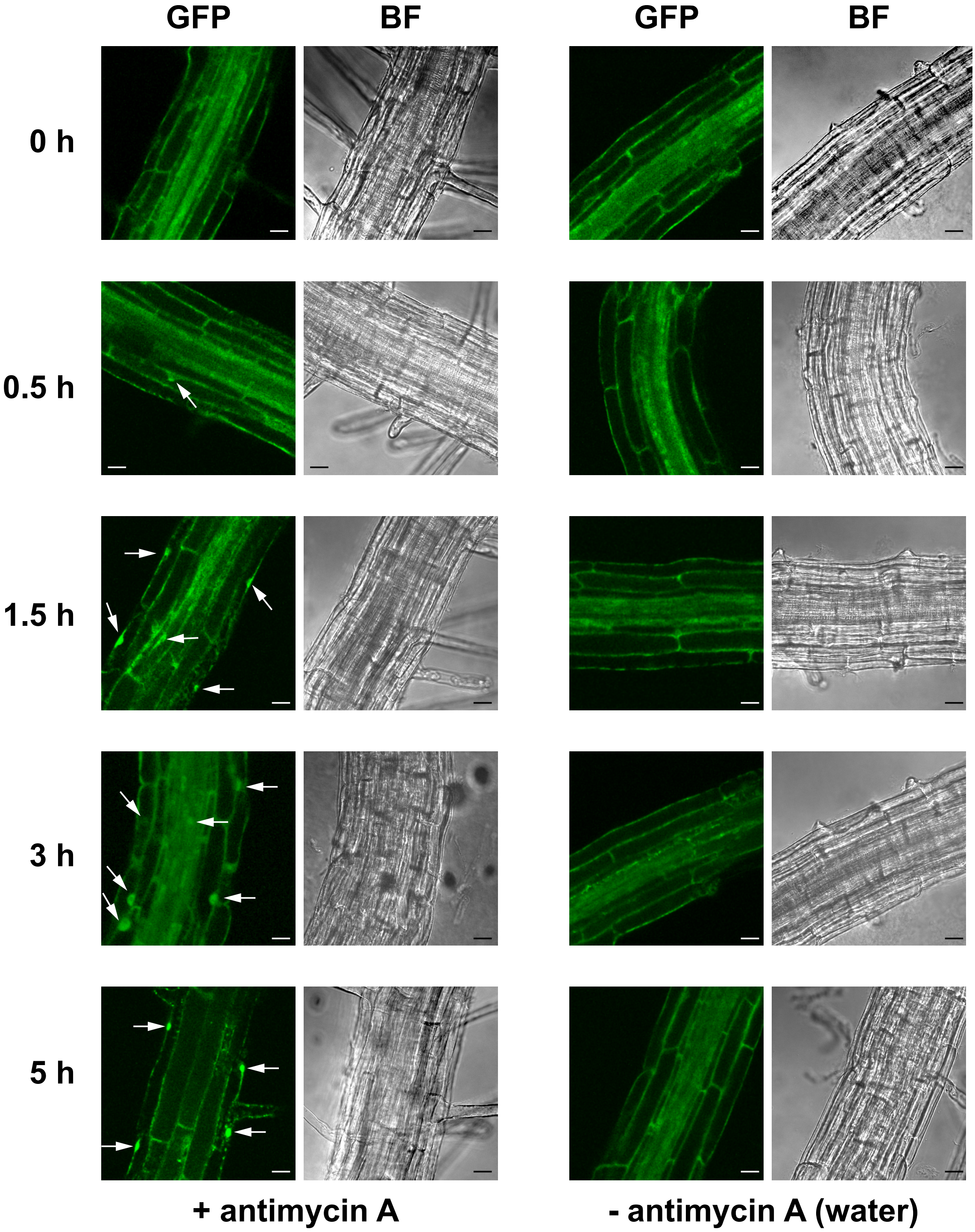

Supplemental Figure 2: Accumulation of an ANAC017-GFP fusion protein in the nucleus after antimycin A treatment (supports Figure 3).

A transgenic line expressing an ANAC017-GFP fusion protein under the control of the cauliflower 35S promoter was treated with antimycin A or water. The subcellular distribution of fluorescence was subsequently monitored over 5 h using a Zeiss LSM780 confocal microscope. Accumulation of GFP signal in the nucleus was apparent 1.5 h post-treatment in most cells, indicating release of ANAC017-GFP protein from the ER and its translocation to the nucleus. Shown are representative images for GFP detection (excitation: 488 nm, emission: 490-600 nm) and bright field (BF). White arrows indicate nuclei and scale bars 20 µm.

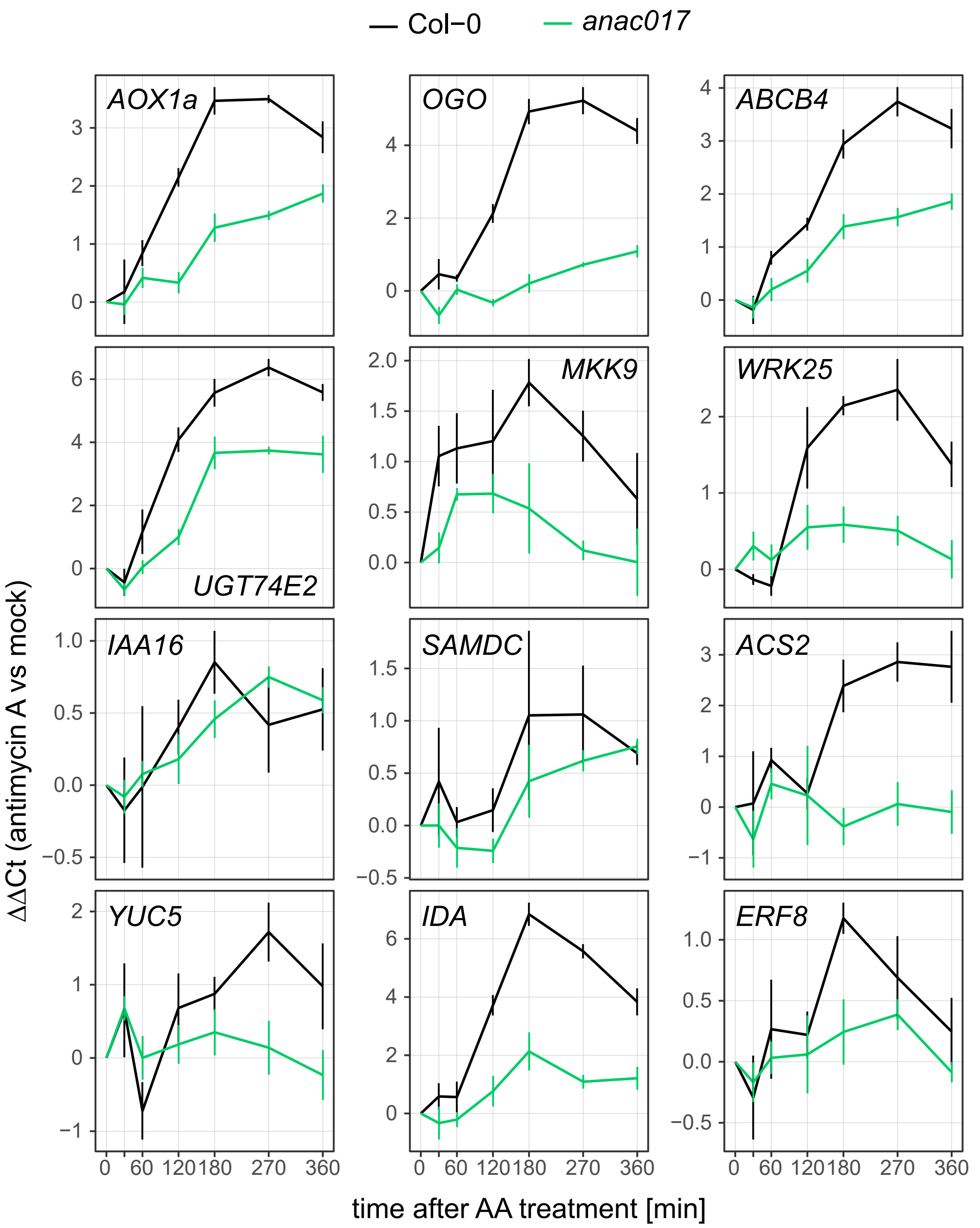

Supplemental Figure 3: Induction of ANAC017 target genes is attenuated in an *anac017* mutant line (supports Figure 6)**.**
Activation of genes binding ANAC017 as demonstrated by ChIP-seq (see Figure 6A, B) was attenuated in the *anac017* mutant line. Wild type (Col-0) and the *anac017* lines were sprayed with AA or water and gene expression quantified by RT-qPCR at the given time points. Given are the means of the ΔΔCt ± SE values (AA vs. control, n = 3).
