# Supplementary material for: The retrograde signaling regulator ANAC017 recruits the MKK9–MPK3/6, ethylene, and auxin signaling pathways to balance mitochondrial dysfunction with growth
Source: Plant Cell. 2022 Jun 16;34(9):3460–81. doi: 10.1093/plcell/koac177 (PMC9421482; doi:10.1093/plcell/koac177)
Supplement: koac177_Supplementary_Data [file tpc22.00293.PeerReview.pdf › tpc22.00293.PeerReview.pdf]

The retrograde signalling regulator ANAC017 recruits the MKK9-MPK3/6, ethylene, and auxin signalling pathways to balance mitochondrial disfunction with growth

Cunman He, Lim Chee Liew, Lingling Yin, Mathew G. Lewsey, James Whelan, Oliver Berkowitz

Corresponding author: Oliver Berkowitz o.berkowitz@latrobe.edu.au.

Review timeline:

TPC2022-RA-00105	Submission received:	Jan. 28, 2022
	1st Decision:	March 24, 2022 *manuscript declined*
**TPC2022-RA-00293**	Submission received:	May 18, 2022
	1st Decision:	May 29, 2022 *acceptance pending, sent to science editor*
	Final acceptance:	June 9, 2022

REPORT: (The report shows the major requests for revision and author responses. Minor comments for revision and miscellaneous correspondence are not included. The original format may not be reflected in this compilation, but the reviewer comments and author responses are not edited, except to correct minor typographical or spelling errors that could be a source of ambiguity.)

TPC2021-RA-00105 1st Editorial decision – *declined* **March 24, 2022**

The reviewers agreed that your work illustrates the critical role of retrograde mitochondrial signaling in the regulation of MAP kinase, ethylene, and auxin signaling. However, the editors (in their pre-review consultation session) and Reviewer #1 raised several concerns which will need to be addressed during revision to better support the main conclusions of your manuscript, including the conclusions drawn based on the transcriptomic data and the ChIP-seq dataset. It will also be essential to demonstrate the relevance of the changes in the expression levels of auxin- and ethylene-responsive genes in the *anac107* mutant relative to wild-type plants (including the associated plant phenotypes) under conditions that induce retrograde mitochondrial signaling.

-- Reviewer comments:

Reviewer #1 (Comments for the Author):

Retrograde signals from mitochondria were reported to participate in various stress response in plants and animals. In plants, the phytohormone ethylene was shown to has an important function in MRR (mitochondria retrograde response), however, the regulatory network remains largely unknown. In this manuscript, the authors established a model about how the plant cells response to mitochondria dysfunction induced by antimycin A treatment, and illustrate the key role of ANAC17 TF and the downstream ethylene and auxin signaling in this process. This work will largely broad our knowledge about the mitochondria retrograde signaling, and the interplay between MRR and phytohormone signaling and their roles in stress response. I have several concerns which I list below:

1. In Fig 3C and line 215-216, the authors claim that the cluster 1-3 genes "included the DEGs with limited changes in expression in response to the two treatments". From my side, the cluster 2 and 3 genes are largely repressed for AA treatment in WT and *ogo-1*, but attenuated in *ein3*.
2. Line 217-219, the cluster 4 genes were defined as touch induced genes and not related to AA treatment. However, the response of these genes in AA treated WT and ago1 is much stronger than untreated samples, especially at 30 min time point, the untreated sample has negative response, but very strongly induced in treated samples. I wonder if these genes are very early AA response genes. Specially, in Fig.5, the ethylene-related genes *ERF042*, *ERF019* and *GGT1* and auxin synthesis gene *YUC5* have the similar pattern with cluster 4 genes, but was discussed as important early response genes for ethylene/auxin response to AA treatment. I suggest the author to dig into the cluster 4 genes to elevate their contribution in Mito dysfunction response.
3. In Fig. 4, the DREM analysis gives an impressive clue about the important function of ANAC17 (and ANAC13) in the MRR, which in *ein3* mutant is somehow attenuated. I wonder what happens in *ogo1* mutant?
4. The auxin response was discussed to be at later stages of MRR, from 120 min the induction of negative factors in

auxin signaling and auxin conjugation enzyme to repress the auxin response and plant growth because the plants try to defense the stress. Later, the auxin synthesis was enhanced (induction of auxin synthesis genes), which the authors discussed as to repress the MRR. However, at very early stages (30, 60min) the auxin synthesis gene *YUC5* (ANAC17 directly binding) and signaling gene *RCE1*, *PBP1* were quickly induced, I wonder what the possible role of this auxin induction in MRR (as early as ethylene response) is.

5. In the *ein3* mutant, the response of auxin-related genes was largely repressed. The authors claimed that the auxin response is somehow late than and dependent on ethylene signaling. The ANAC17 could bind to the promoter of auxin related genes (YUC5, ABCB4, IAA16 and UGT74E2). I wonder if the ANAC17 regulated auxin genes are the early layer of auxin response in MRR, and the ethylene regulated auxin genes represent the late response auxin genes, since the authors discussed in the "Discussion" that ANAC17 is even upstream of ethylene. However the ABCB4 and UGT74E2 only upregulated at late stage (120/180min).

6. ANAC17 binds and active the auxin and ethylene genes shown by ChIP and in OE line by RT-qPCR (Fig.6). To make this conclusion more convincing, the response of these genes in AA treatment in anac17 mutant should be analyzed.

7. In Fig.7E, the model, the authors concluded that ANAC17 is upstream of ethylene (and auxin), mainly based on the finding that the ANAC17 can bind the promoter of ethylene synthesis (ACS2, SAMDC) and response (ERF8, MKK9, IDA) genes. The MPK signaling cascade was reported to be the very first/upstream (response in minutes) event in many stress signaling. The upregulation of MKK9 by ANAC17 could be an amplification of the MPK/ethylene signaling in MRR. It was shown in Ng et al, 2013, the ANAC17 was necessary for >85% of the changes observed as a primary response to cytosolic H_2O_2. It seems that ANAC17 is a general regulator of cellular oxidative stress. Its function in AA treatment maybe later than MPK signaling. I wonder how fast that the ANAC17 will be cleaved and moved to nucleus in response to AA treatment. In cluster 5, the protein phosphorylation was enriched, if it is possible that the cleavage of ANAC17 is dependent on the phosphorylation (ANAC17 or the rhomboid protease) by MPK?

Minor points:

1. Line 190, antimycin A, the abbreviation should (have to) be defined when it is first used in the manuscript.
2. In Fig.2A-C, the statistical analysis should be performed to see if the changes in qPCR are significant.
3. The author should check their reference list, for example, line 756, 769, the volume and page number were missing; some journals were in full name, but some are with abbreviations, and so on. The authors should check carefully to avoid these issues.

Reviewer #2 (Comments for the Author):

This work shows that the transcription factor ANAC017 directly controls important genes of the ethylene and auxin signalling pathways, including the MAP kinase kinase MKK9 and the key gene for ethylene biosynthesis, ACS2 as well as the auxin response gene YACCA 5. Moreover, also other transcription factors that regulate plant growth and stress responses, such as the bZIP transcription factors bZIP60 and bZIP53 or ANAC081 and RADICAL-INDUCED CELL DEATH1 were found to be targeted by ANAC017, based on Chromatin immunoprecipitation followed by sequencing (ChIP-seq) and overexpression analysis. Dynamics of the responses was studied by RNA-seq, showing that ethylene signalling precedes the stimulation of auxin signalling in the mitochondrial stress response, and a large part of the transcriptional regulation was dependent on ETHYLENE INSENSITIVE 3. These findings were further confirmed by mutant analyses. In summary, this work identifies the molecular components which are controlled by ANAC017 and integrate primary plant stress responses and mitochondrial dysfunction with whole plant growth.

The fact that mitochondria relay their status to the nucleus via a pathway termed mitochondrial retrograde response (MRR), which adjusts the expression of nuclear genes to optimize mitochondrial function is known for many years. Also, the Arabidopsis NAC domain transcription factor ANAC017 was identified as convergence point in organellar signalling. However, a detailed knowledge on direct targets of these signalling pathways and a mechanistic understanding was so far lacking. Recently connections between auxin and ethylene signalling and the MRR were discovered, and interestingly, also in the chloroplast retrograde response, these connections seem to emerge. Thus, underpinning the notion that indeed ANAC017 could be part of convergence in retrograde organellar signalling. Accordingly, the evidence provided here that ANAC017 activates ethylene and auxin signalling in parallel with the

MKK9-MPK3/6 MAP kinase pathway is an important step towards mechanistical understanding of retrograde organellar signalling.

The study is based on a broad set of different transcriptional studies analysing expression of genes for mitochondrial metabolism and effects of different stress treatments or physiological conditions including also different reporter lines and mutants. This combination, which is used to test the connections between the MRR pathway and the different hormone and MAP kinase signalling pathways, makes the unique strength of this study and, together with a detailed kinetic analysis of the signalling events, provide novel insights in the connections between them.
The data are very solid and include a stringent and careful statistical analysis. It is striking to see the clear outcome of this analysis, pointing out almost exclusively these targets that are key factors in regulating those signalling pathways, which have been somehow connected to retrograde signalling but were never put together in this context so clearly, and have not been studied in this context so systematically. Subsequently, these connections were further confirmed by qPCR analysis of target genes and binding of ANAC017 to the promoters of target genes was determined by ChIP-seq experiments using a transgenic plant line after inducing mitochondrial stress by spraying with antimycin A (AA) or myxothiazol (MT). This led to the finding that ANAC017 directly binds to the promoters of MKK9 and ACS2, potentially explaining how an increase in ethylene biosynthesis could be achieved to promote the early mitochondrial stress response.

The presentation of these very complex data sets is very well-thought-out and does nicely lead to the important key points. Usually, I have my problems with seeing immediately the biology behind so many gene expression studies and network analyses. Here, the results do guide the reader intuitively to the involved biological pathways.

Points for revisions:

I am really sorry for this I have been thinking for a while now but I don't find anything. Usually that does not happen.

Reviewer #3 (Comments for the Author):
The work "THE RETROGRADE SIGNALING REGULATOR ANAC017 RECRUITS THE MKK9-MPK3/6, ETHYLENE AND AUXIN SIGNALING PATHWAYS TO BALANCE MITOCHONDRIAL DYSFUNCTION WITH GROWTH" represents a hallmark piece of evidence and an important advance in elucidating the mechanisms that govern the interaction of the mitochondrial activity with the cellular energy demands to coordinate growth and stress responses in plants. The results help to elucidate the mechanisms involved in the MRR processes. Identifying, for the first one, the genes and protein factors involved downstream of the key and protein ANAC017.
In this work, the interaction between mitochondrial dysfunction and the hormonal pathways that regulate growth and response to stress in plants is analysed. The chronology of the events is established for the first time, highlighting a first response mediated by ethylene, and a later one in which the central role of auxins is marked.
Here, a central regulator of the response to mitochondrial dysfunction, the transcription factor ANACO17, has a central role, in regulating the expression of MAKK 9, which in turn participates in the phosphorylation of factors significant for the synthesis of ethylene and its subsequent mechanism of action.
The work is brilliant, there are no fissures. Every question this reviewer asked was answered by the authors in the following section. A detailed bioinformatic analysis, accompanied by the corresponding experimental corroboration. The figures are clearly presented, with appropriate references that help to understand the experimental logic and the results obtained.

TPC2022-RA-00293 Submission received May 18, 2022

AUTHOR'S RESPONSE TO REVIEWING EDITOR: We appreciate the overwhelming positive feedback from reviewer 2 ("I can't think of any revisions") and reviewer 3 ("The work is brilliant, there are no fissures."), with both suggesting no revisions. Reviewer 1 was also very positive about the importance and broader implications to our understanding of mitochondrial function and plant growth. We have addressed all concerns where necessary with further experiments or added clarification within the text as outlined below.

Especially in response to the editor's comment and point 6 of reviewer 1, we have conducted time-course RT-qPCR on genes related to ethylene/auxin and directly targeted by ANAC017, as determined by ChIP-seq (Figure 6B) in an

anac017 mutant. Consistent with data presented in our previous submission, the induction of these genes after AA treatment is reduced, in agreement with earlier results from our lab (Ng et al, Plant Cell 2013). Hence these results confirm the regulation of these genes by ANAC017.

Reviewer comments on previously declined manuscript and **author responses:**

Reviewer #1:

Point 1. In Fig 3C and line 215-216, the authors claim that the cluster 1-3 genes "included the DEGs with limited changes in expression in response to the two treatments". From my side, the cluster 2 and 3 genes are largely repressed for AA treatment in WT and *ogo-1*, but attenuated in *ein3*.

RESPONSE: The reviewer is correct in the description of cluster 2 and 3 genes, i.e. their down-regulation after AA treatment. We have now added reference to cluster 2 and 3 to the description of those clusters (L195-196). We believe highlighting here the more 'classical' dysfunction response of clusters 5 and 6 is warranted to validate the experiment and highlight the new findings of ethylene involvement and EIN3 dependency. Clusters 2 and 3 genes were described in more detail in the DREM model, i.e. the photosynthesis related genes, and we tried to avoid this redundancy. We have added reference to cluster 2 and 3 to the section describing this particular part of the DREM model (L298) to more strongly emphasis this relation between clusters and DREM paths.

Point 2. Line 217-219, the cluster 4 genes were defined as touch induced genes and not related to AA treatment. However, the response of these genes in AA treated WT and *ago1* is much stronger than untreated samples, especially at 30 min time point, the untreated sample has negative response, but very strongly induced in treated samples. I wonder if these genes are very early AA response genes. Specially, in Fig.5, the ethylene-related genes *ERF042*, *ERF019* and *GGT1* and auxin synthesis gene *YUC5* have the similar pattern with cluster 4 genes, but was discussed as important early response genes for ethylene/auxin response to AA treatment. I suggest the author to dig into the cluster 4 genes to elevate their contribution in Mito dysfunction response.

RESPONSE: We agree that some of the cluster 4 genes might also have a function in the mitochondrial stress response and/or AA treatment might accentuate their response to water spraying. This is agreement with earlier findings from members of our team that show that mitochondrial function modulates touch signalling (van Aken et al Plant Physiol (2016). 171: 2150-2165, Xu et al, Plant J (2019). 97: 623-645) and that water spraying alone activates a signalling cascades involving MYC2 (Van Moerkercke et al., PNAS 116, 23345-23356 (2019), Matsumura et al, Nature Communications 2022, 13:2016). Correspondingly, MYC2 is also a member of cluster 4. These interactions are very difficult to entangle and would have to be done on a gene-by-gene basis. GO termed enrichment analysis for cluster 4 however only identified microbial defence responses that involves salicylic and jasmonic acid signalling, but no GO terms directly attributable to mitochondrial function, antimycin A effects nor auxin or ethylene signalling (Supplemental Table 4). This is in agreement with above cited publications and in line with the response to water spray involving MYC2.

Just to clarify, we would like to point out that the response is not negative as the reviewer suggests, the scale represents a z-scored expression, i.e. a negative value indicates an expression lower than the median.

Point 3. In Fig. 4, the DREM analysis gives an impressive clue about the important function of ANAC17 (and ANAC13) in the MRR, which in *ein3* mutant is somehow attenuated. I wonder what happens in *ogo1* mutant?

RESPONSE: We have added the DREM model for _ogo-1_ to Figure 4 for completeness and its description to the Results section (L377-386). Briefly, the DREM model also shows ANAC017 as the major regulator of the up-regulated paths and a down-regulation of photosynthesis-related genes as observed in Col-0.

Point 4. The auxin response was discussed to be at later stages of MRR, from 120 min the induction of negative factors in auxin signaling and auxin conjugation enzyme to repress the auxin response and plant growth because the plants try to defense the stress. Later, the auxin synthesis was enhanced (induction of auxin synthesis genes), which the authors discussed as to repress the MRR. However, at very early stages (30, 60min) the auxin synthesis gene *YUC5* (ANAC17 directly binding) and signaling gene *RCE1*, *PBP1* were quickly induced, I wonder what the possible role of this auxin induction in MRR (as early as ethylene response) is.

RESPONSE: Our manuscript is the first to identify and further characterise an interaction of the two hormones with mitochondrial signalling. We believe to completely understand these intricacies will take many more experiments on

a gene-by-gene basis. We thus acknowledge that our analysis will not capture all complexities of these integrated pathways, but it provides the basis for future experiments. Possible additional factors such as tissue-specific expression will also have impact as shown before (Berkowitz et al (2021) Plant J 107: 938-955), as well as the diverse function of genes in the pathway varying from biosynthesis to conjugation. In addition, the interaction for auxin and ethylene are both synergistic and antagonistic depending on the context (Muday et al, Auxin and ethylene: collaborators or competitors? Trends Plant Sci 2012 Vol. 17 Issue 4 Pages 181-95).

To better visualise the overall response of ethylene- and auxin-related genes in response to antimycin A treatment, we have added a panel to Figure 5 (lower panel). This uses the expression of these genes for a regression analysis. This confirms a concerted stronger response of ethylene-related genes earlier than auxin-related genes. See also our response to point 5.

We can only speculate on RCE1 and PBP1 and refrained from doing so in the manuscript. RCE1 is part of the auxin reception pathways and hence could sensitise the cell for increasing auxin levels following subsequent synthesis, while PBP1 has been shown to be part of the touch response (Benjamins et al. (2003), Plant Physiol 132, 1623-1630).

Point 5. In the *ein3* mutant, the response of auxin-related genes was largely repressed. The authors claimed that the auxin response is somehow late than and dependent on ethylene signaling. The ANAC17 could bind to the promoter of auxin related genes (YUC5, ABCB4, IAA16 and UGT74E2). I wonder if the ANAC17 regulated auxin genes are the early layer of auxin response in MRR, and the ethylene regulated auxin genes represent the late response auxin genes, since the authors discussed in the "Discussion" that ANAC17 is even upstream of ethylene. However the ABCB4 and UGT74E2 only upregulated at late stage (120/180min).

RESPONSE: We have added a panel to Figure 5 summarising the response of auxin- and ethylene-related genes, showing a concerted, earlier response of ethylene-related genes. This agrees with previous reports from our lab showing that auxin represses the mitochondrial dysfunction response (Ivanova et al. Plant Physiol 2014 Vol. 165 Issue 3 Pages 1233-1254) and inhibitors of auxin signalling activate the MRR (Kerchev et al. Molecular Plant 2014 Vol. 7 Issue 7 Pages 1138-1150). While these overall observations are contrary to the reviewer's suggestion of a generally earlier auxin response, we agree that a few individual genes show an early response. We believe this is not surprising given the complexity of ethylene-auxin interactions that impact hormone homeostasis, but also the range of protein functions (biosynthesis, transport, conjugation) that will likely have varying impact on genes during the course of the response to mitochondrial stress. See also our response to point 4.

Point 6. ANAC17 binds and active the auxin and ethylene genes shown by ChIP and in OE line by RT-qPCR (Fig.6). To make this conclusion more convincing, the response of these genes in AA treatment in *anac17* mutant should be analyzed.

RESPONSE: We have performed RT-qPCR to quantify the expression of these genes in the *anac017* mutant background. As expected from a previous report (Ng et al Plant Cell 2013 Vol. 25 Issue 9 Pages 3450-71), their induction was attenuated after antimycin A treatment. This data were added to the resubmission as Supplemental Figure 3.

We would like to point out that because of some functional redundancy of ANAC017 with other TFs of this family, esp. ANAC013, a complete abolishment of the AA response is not to be expected. We therefore believe the upregulation of these genes after ANAC017 overexpression together with the ChIP-seq results are more direct evidence for the regulation of these genes by ANAC017.

Point 7. In Fig.7E, the model, the authors concluded that ANAC17 is upstream of ethylene (and auxin), mainly based on the finding that the ANAC17 can bind the promoter of ethylene synthesis (*ACS2*, *SAMDC*) and response (*ERF8*, *MKK9*, *IDA*) genes. The MPK signaling cascade was reported to be the very first/upstream (response in minutes) event in many stress signaling. The upregulation of MKK9 by ANAC17 could be an amplification of the MPK/ethylene signaling in MRR. It was shown in Ng et al, 2013, the ANAC17 was necessary for >85% of the changes observed as a primary response to cytosolic H_2O_2. It seems that ANAC17 is a general regulator of cellular oxidative stress. Its function in AA treatment maybe later than MPK signaling. I wonder how fast that the ANAC17 will be cleaved and moved to nucleus in response to AA treatment. In cluster 5, the protein phosphorylation was enriched, if it is possible that the cleavage of ANAC17 is dependent on the phosphorylation (ANAC17 or the rhomboid protease) by MPK?

RESPONSE: We have used a transgenic line expressing a ANAC017-GFP fusion protein and treated it with AA.

Accumulation of ANAC017-GFP in the nucleus was first visible after 30 min of treatment in a few cells and then becoming increasingly wide-spread after 1.5 h (this data was added to the manuscript as Supplemental Figure 2 and L225229). This fits the kinetics of the RNA-seq experiment, esp. genes in cluster 5 and 6. This is also consistent with the activation of MPK3 and MPK6 occurring around 2 h after oxygen deprivation, antimycin A and myxothiazol treatment (Chang et al, Plant Mol Biol 2012 Vol. 78 Issue 1-2 Pages 109-22). Together these results show the activation of MPKs after antimycin A treatment is not within minutes but rather within the time frame of ANAC017 accumulation in the nucleus and up-regulation of genes (cluster 5 and 6). We added a sentence to the Discussion to point this out more clearly (L578-581).

The reviewer's suggestions are valid and a good proposal for further investigation in the future. The idea of an amplification cycle is interesting. However, we show that the upregulation of ACS2 (a target of MPK3 and MPK6) and MKK9 (an activator of MPK3 and MPK6) is driven by ANAC017. This suggests ANAC017 is upstream of both.

We feel without further evidence of ANAC017 phosphorylation or targets of MPKs involved in the mitochondrial signalling pathway (the AA response is not abolished in the *mpk3 mpk6* mutant background), this would add unwarranted speculation.

<u>Reviewer #2:</u>

Point 1. I am really sorry for this I have been thinking for a while now but I don't find anything. Usually that does not happen.

RESPONSE: We highly appreciate the reviewer's positive comments.

<u>Reviewer #3:</u>

Point 1. The work is brilliant, there are no fissures. Every question this reviewer asked was answered by the authors in the following section. A detailed bioinformatic analysis, accompanied by the corresponding experimental corroboration. The figures are clearly presented, with appropriate references that help to understand the experimental logic and the results obtained.

RESPONSE: We are delighted by the overwhelming positive feedback.

TPC2022-RA-00293 1st Editorial decision – *acceptance pending* May 29, 2022

We are pleased to inform you that your paper entitled "THE RETROGRADE SIGNALLING REGULATOR ANAC017 RECRUITS THE MKK9-MPK3/6, ETHYLENE AND AUXIN SIGNALLING PATHWAYS TO BALANCE MITOCHONDRIAL DYSFUNCTION WITH GROWTH" has been accepted for publication in The Plant Cell, pending a final minor editorial review by journal staff. At this stage, your manuscript will be evaluated by a Science Editor with respect to its presentation of scientific content, compliance with journal policies, and presentation for a broad readership.

Final acceptance from Science Editor June 9, 2022
